# Simulated Warming Alters Soil Nutrient Dynamics Through Microbial Community Shifts During Wetland Plant Litter Decomposition

**DOI:** 10.3390/microorganisms14102320

**Published:** 2026-10-12

**Authors:** Jiaxin Wang, Guangying Zhao, Qing Xu, Lijuan Zhang, Xingfeng Dong

**Affiliations:** 1 College of Geographical Science, Harbin Normal University, Harbin 150025, China; 17519510781@163.com (J.W.); 17703270849@163.com (Q.X.); zhlj@hrbnu.edu.cn (L.Z.); 2 Heilongjiang Province Key Laboratory of Geographical Environment Monitoring and Spatial Information Service in Cold Regions, Harbin 150025, China; 3 Huzhong Permafrost and Cold Environments Observation and Research Station of Heilongjiang Province, Da Hinggan Ling 165036, China

**Keywords:** climate warming, wetland soil, litter decomposition, nutrient cycling, microbial community

## Abstract

Plant litter decomposition drives soil biogeochemical cycling, yet how warming alters interactions among litter quality, microbial communities, and soil C, N, and P transformations remains unclear. We conducted a 90-day soil microcosm incubation at 15, 25, and 35 °C using single and mixed litter of *Calamagrostis angustifolia* and *Carex lasiocarpa* from the Xingkai Lake wetland to assess warming effects on decomposition, microbial communities, and C, N, and P dynamics. Warming significantly accelerated decomposition, with the highest decomposition constant observed for mixed litter at 35 °C. Mixed litter showed greater temperature sensitivity at 25–35 °C, whereas decomposition rates were weakly correlated with initial litter C/N, C/P, and N/P ratios. Warming and decomposition time reshaped microbial communities, with bacterial diversity increasing over time and fungal diversity declining at 35 °C. Despite accelerated mass loss, C, N, and P dynamics remained asynchronous, with net C release in some treatments, net N accumulation, and litter-dependent P responses. Three-way ANOVA, RDA, and partial least squares structural equation modeling were used to examine associations among environmental factors, microbial attributes, litter decomposition, and soil C, N, and P status. Overall, warming modifies relationships among litter quality, microbial communities, and C, N, and P transformations, providing insights into freshwater wetland responses to climate warming.

## 1. Introduction

Global warming has become one of the most important drivers altering terrestrial ecosystem processes by regulating soil biogeochemical cycling. As a highly climate sensitive component of the terrestrial carbon pool, high-latitude wetlands store large amounts of soil organic carbon (SOC) and are particularly vulnerable to rising temperatures [1,2,3]. In these ecosystems, plant litter decomposition links aboveground primary productivity with belowground carbon and nutrient cycling [4,5]. Although warming generally accelerates litter decomposition by stimulating microbial metabolism and extracellular enzyme activity, decomposition rates are jointly regulated by litter substrate quality, microbial communities and soil environmental conditions [6]. In wetland soils, high moisture and strong soil-litter contact may alter the relative importance of litter substrate quality and microbial regulation controls on decomposition [7]. Therefore, understanding whether warming shifts the dominant controls of litter decomposition is essential for predicting wetland soil biogeochemical responses under future climate change.

Substrate quality has long been regarded as a key factor shaping the response of litter decomposition to warming [8]. Litter C/N, C/P and N/P ratios influence nutrient availability for decomposer microorganisms and are therefore commonly used to explain variation in decomposition rates and nutrient release [9]. The substrate quality–temperature hypothesis further predicts that nutrient-poor or chemically recalcitrant substrates may show greater temperature sensitivity because their decomposition requires higher activation energy [10]. Accordingly, warming effects on decomposition may differ among plant species and litter mixtures with contrasting chemical traits. However, the expected relationship between initial litter quality and the temperature sensitivity of decomposition is not always consistent across ecosystems. Microbial acclimation, background nutrient availability, and local soil conditions can weaken or even obscure the extent to which initial litter chemistry predicts decomposition responses to warming [11]. Much of the evidence supporting substrate-quality control comes from forests and grasslands, where decomposition generally occurs in relatively well-drained soils [12]. Wetland soils differ markedly from these systems. Prolonged water saturation, close soil–litter contact and active nutrient exchange may buffer the stoichiometric constraints imposed by litter chemistry [13]. Whether initial litter stoichiometry remains a dominant control of decomposition under warming in high-moisture wetland soils therefore remains unresolved.

Soil microorganisms are central to litter decomposition and nutrient transformation because they mediate the depolymerization, assimilation and immobilization of litter-derived C, N and P before these elements enter soil nutrient pools [14,15]. Changes in microbial communities can therefore affect not only litter mass loss, but also the balance between nutrient release and immobilization during decomposition. Warming may modify these processes by increasing microbial metabolic demand, altering the relative roles of bacterial and fungal decomposers, and selecting taxa with different substrate-use strategies [16]. Bacteria generally respond rapidly to labile substrates, whereas fungi are often more involved in degrading structurally complex plant residues and may be more sensitive to thermal stress [17]. Beyond community composition and diversity, microbial association patterns may also shift during decomposition. Although co-occurrence networks do not provide direct evidence of biological interactions, they can help describe microbial community organization under different temperature and substrate conditions [18]. Linking microbial community shifts and network reorganization with litter C, N and P dynamics is therefore important for understanding how warming regulates soil nutrient transformation during wetland litter decomposition.

Litter species composition may further modify warming effects on decomposition and nutrient release. In natural wetlands, plant residues usually enter soils as mixed-species litter rather than as single-species inputs. Compared with single-species litter, mixed litter can alter decomposition through resource complementarity, nutrient transfer, and shifts in microbial niche partitioning [19]. These non-additive effects may accelerate or suppress decomposition depending on the chemical traits of the component species and the surrounding soil environment [20]. By enhancing resource complementarity and broadening substrate availability to decomposers, litter mixing may alleviate resource limitation and increase the responsiveness of microbial decomposition to warming. Mixed litter may therefore exhibit greater temperature sensitivity of decomposition than single-species litter, although the magnitude and direction of this response may depend on litter composition and environmental conditions [21]. However, relatively few studies have examined whether litter mixing modifies decomposition temperature sensitivity and the associated C, N, and P dynamics in wetland soils [22]. This gap limits our ability to predict litter-driven soil biogeochemical responses in plant communities where multiple litter types decompose simultaneously.

The Xingkai Lake wetland in Northeast China provides a suitable system for examining these questions because it is a high-latitude freshwater wetland with organic-rich soils and dominant macrophyte communities [23]. The region experiences strong seasonal temperature variation and is sensitive to ongoing climate warming, making it an appropriate system for investigating temperature effects on litter decomposition and associated soil C, N, and P cycling. *Calamagrostis angustifolia* and *Carex lasiocarpa* are common wetland macrophytes in this region and produce litter with contrasting chemical traits, providing an opportunity to compare single-species and mixed-litter decomposition under warming. In this study, we conducted a 90-day microcosm incubation using litterbags embedded in wetland soil under three temperature regimes: 15, 25, and 35 °C. We aimed to determine: (1) how warming affects litter decomposition rates and C, N, and P dynamics; (2) whether initial litter stoichiometry remains a dominant predictor of decomposition across contrasting litter types and whether litter mixing modifies this relationship in high-moisture wetland soil; and (3) how shifts in microbial communities and network organization are associated with soil nutrient transformations during decomposition. We hypothesized that warming would accelerate litter decomposition while weakening the influence of initial litter stoichiometry, that mixed litter would exhibit greater decomposition temperature sensitivity than either single-species litter treatment, and that microbial community and network reorganization would be associated with asynchronous C, N, and P transformations in wetland soils.

## 2. Materials and Methods

### 2.1. Study Site Description

**Figure 1:**
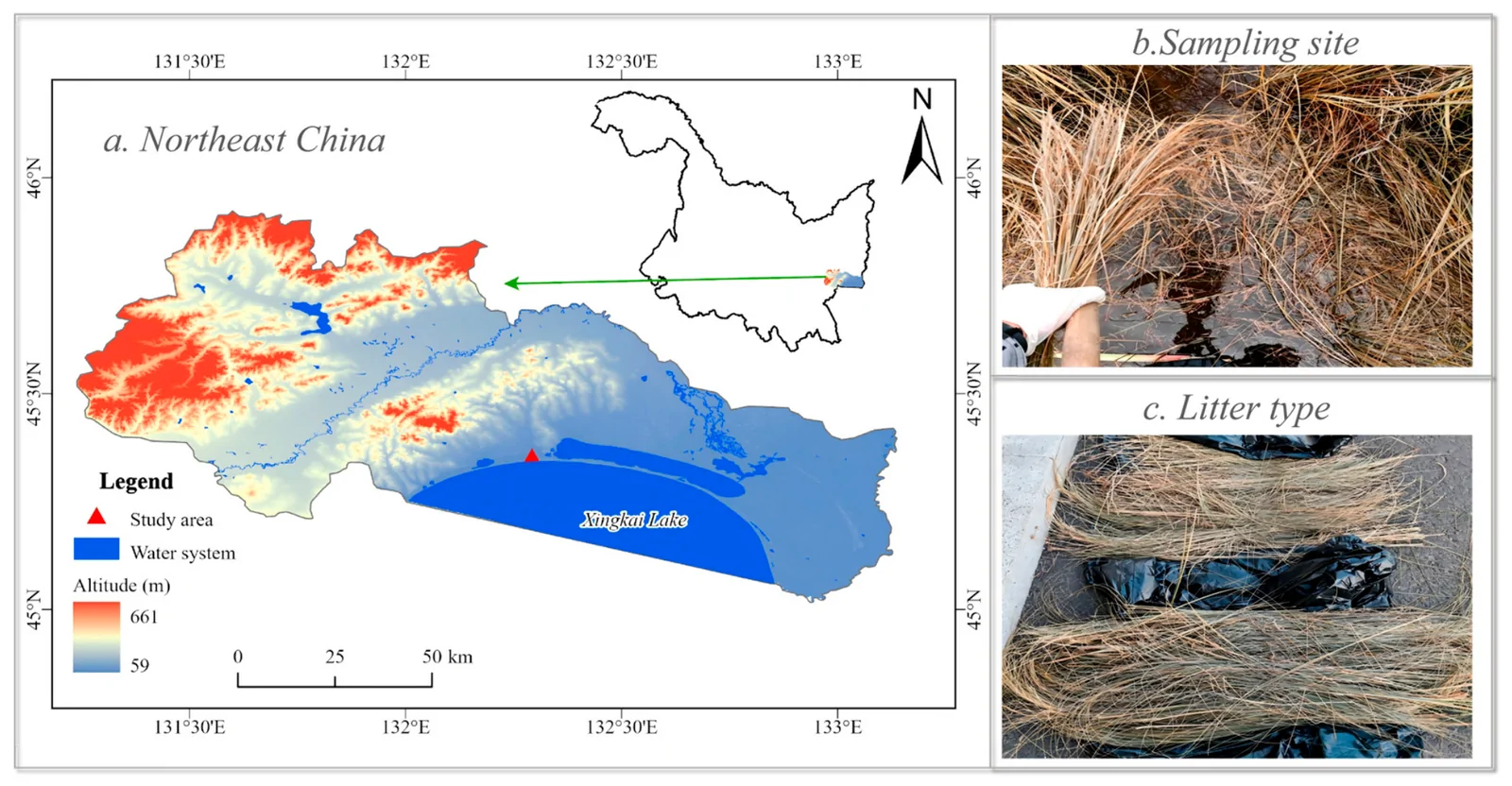
Location of the study area, sampling site and litter materials collected for the experiment. (**a**) Location of Xingkai Lake in Northeast China; (**b**) the sampling site; and (**c**) litter collected from the plant species used in the experiment, *C. angustifolia* (upper) and *C. lasiocarpa* (lower).

Xingkai Lake (approximately 44°32′–45°21′ N, 131°58′–132°51′ E) is a transboundary freshwater lake located along the China–Russia border in Northeast Asia [24]. On the Chinese side, the Xingkai Lake National Nature Reserve is designated as a Ramsar Wetland of International Importance [25]. The sampling site was located at 45.35704943° N, 132.292625° E (Figure 1). The region has a temperate continental monsoon climate characterized by warm, humid summers and long, cold winters, with a mean annual temperature of approximately 3.0 °C and a mean annual precipitation of approximately 654 mm. The lake surface remains frozen for approximately 180 d each year, and precipitation occurs predominantly during the warm season [23]. Swamp soils predominate in the reserve [22]. At the sampling site and in nearby plots, the wetland soils are classified as Histosols under the World Reference Base for Soil Resources (WRB; IUSS Working Group WRB, 2022 [26]) and are characterized by abundant organic matter and root remains at different stages of decomposition. *C. angustifolia* and *C. lasiocarpa* are representative wetland species in Northeast China [27]. Field observations at the sampling site indicated that the plant community was dominated by *C. angustifolia*, with *C. lasiocarpa* occurring sporadically as an associated species. Soil and naturally senesced plant litter used in the microcosm experiment were collected from this sampling site.

### 2.2. Sample Collection and Processing

In October 2025, surface soil and naturally senesced plant litter were collected from a sampling site (45.35705° N, 132.29263° E) in the Xingkai Lake wetland. The surface litter layer was removed as a whole, and the exposed soil surface served as the reference for sampling depth. Five bulk subsamples of organic soil material were then collected over the full 0–20 cm interval at the four corners and center of a square sampling plot using a clean stainless-steel shovel. The five subsamples were pooled and homogenized to form a composite soil sample [28]. The composite soil was transported to the laboratory on the day of collection. Visible gravel, coarse roots, and plant debris were manually removed, after which the fresh soil was passed through a 2-mm sieve and stored at 4 °C until microcosm establishment [29]. All 81 experimental microcosms were established using subsamples of the same homogenized composite soil, with 300 g of fresh soil added to each microcosm and three replicates established for each treatment combination [30].

Undisturbed soil cores were collected separately from the same sampling plot using the cutting-ring method for determination of maximum water-holding capacity (MWHC). MWHC was determined gravimetrically using a saturation–drainage procedure. The undisturbed soil cores were saturated with deionized water by capillary wetting, allowed to drain freely under gravity, and then weighed, with MWHC expressed as the mass of retained water relative to oven-dry soil mass [31]. Naturally senesced foliar litter of *C. angustifolia* and *C. lasiocarpa* was collected from the same sampling area. The litter was briefly rinsed with ultrapure water to remove adhering soil particles and visible debris, oven-dried at 65 °C to constant mass, and stored in desiccators until litterbag preparation [32].

### 2.3. Experimental Design

To simulate the diverse thermal regimes decomposing plant litter encounters in the Xingkai Lake wetland, three incubation temperatures were established: 15, 25, and 35 °C [33]. Specifically, the 15 °C treatment served as the ecological baseline, characterizing the ambient topsoil microenvironment during the growing season without warming perturbations. The 25 °C treatment represented growing-season high-temperature phases and served as a proxy for moderate warming scenarios. In contrast, the 35 °C treatment simulated extreme heatwaves or acute, short-term thermal stress. This enabled a systematic evaluation of threshold responses in litter decomposition kinetics and microbial community restructuring under projected extreme climatic events.

Naturally senesced foliar litter of *C. angustifolia* and *C. lasiocarpa* was oven-dried at 65 °C to constant mass and cut into approximately 1-cm-long segments. Three litter treatments were prepared on a dry-mass basis: *C. angustifolia* litter (3.0 g), *C. lasiocarpa* litter (3.0 g), and a 1:1 mixed-litter treatment containing 1.5 g of each species. Each 3.0-g litter sample was enclosed in a nylon litterbag measuring 8 cm × 7 cm with a 0.01-mm mesh size [30].

Experimental microcosms consisted of polyvinyl chloride (PVC) cylinders (9-cm inner diameter, 15-cm height), each containing 300 g of sieved fresh topsoil with a single litterbag embedded horizontally in the upper soil layer. The experiment comprised three litter treatments, three incubation temperatures (15, 25, and 35 °C), and three destructive harvest times (20, 50, and 90 d) [34], with three replicate microcosms for each litter × temperature × harvest-time combination, yielding a total of 81 microcosms. One constant-temperature incubator was assigned to each temperature treatment. Soil moisture was maintained at 100% of the MWHC across all microcosms by gravimetrically replenishing deionized water every 2 d [35]. This provided a controlled high-moisture background for comparing litter decomposition and nutrient dynamics across the experimental treatments. At each harvest, the remaining litter and corresponding soil were collected for determination of their C, N, and P contents. For microbial community analysis, soil subsamples from the three replicate microcosms within each litter × temperature × harvest-time combination were pooled into a single composite sample, yielding 27 composite samples representing all combinations of the three litter treatments, three temperatures, and three harvest times (3 × 3 × 3). These samples were subsequently subjected to high-throughput sequencing.

To evaluate the release and accumulation dynamics of litter total organic carbon (LTOC), total nitrogen (TN), and total phosphorus (TP) during decomposition, the nutrient accumulation index (NAI, %) was calculated as follows [15]:

(1) $$\mathrm{NAI}=\frac{M_{t}\times X_{t}}{M_{0}\times X_{0}}\times 100\%$$

Here, *M*_0_ (g) represents the initial dry mass of the litter; *M_t_* (g) is the remaining dry mass of the litter at sampling time t, corresponding to 20, 50, or 90 d of incubation; *X_t_* (g/kg) is the concentration of total organic carbon, total nitrogen, or total phosphorus in the remaining litter at time *t*; and *X*_0_ (g/kg) is the corresponding initial concentration. An NAI value of less than 100% indicates net nutrient release, whereas an NAI greater than 100% represents nutrient enrichment or biological immobilization.

Litter mass loss over time was fitted to Olson’s negative exponential decay model to calculate the annual decomposition constant *k* [36]:

(2) $$\frac{M_{t}}{M_{0}}=e^{-\mathrm{kt}}$$

Here, *M_t_* and *M*_0_ are defined as in Equation (1); *t* is the decomposition duration expressed in years, calculated by dividing the incubation time in days by 365; and *k* is the annual decomposition constant (yr^−1^). For model fitting, the harvest times of 20, 50, and 90 d therefore corresponded to t = 20/365, 50/365, and 90/365 yr, respectively.

The temperature sensitivity of litter decomposition (*Q*_10_) was calculated from the decomposition constants obtained across different incubation temperatures [37]:

(3) $$Q_{10}={\left(\frac{k_{2}}{k_{1}}\right)}^{\frac{10}{T_{2}-T_{1}}}$$

where *k*_1_ and *k*_2_ are the decomposition constants at temperatures *T*_1_ and *T*_2_ (°C), respectively. Because adjacent temperature treatments differed by 10 °C, *Q*_10_ was calculated separately for the 15–25 °C and 25–35 °C intervals to assess whether the temperature sensitivity of litter decomposition differed between the lower- and higher-temperature ranges.

### 2.4. Analytical Methods

Upon retrieval, the remaining litter was gently rinsed with deionized water to remove adhering soil particles and extraneous debris, and then oven-dried at 65 °C to constant mass. After the constant dry mass was recorded, the litter was ground and passed through a 100-mesh (0.15-mm) sieve for subsequent chemical analyses [38]. Soil samples were homogenized, and aliquots designated for physicochemical analyses were air-dried, ground, and passed through a 100-mesh (0.15-mm) sieve. For microbial community analysis, the fresh soil subsamples collected from the three replicate microcosms within each litter × temperature × harvest-time combination were pooled into one composite sample. Thus, 27 composite soil samples representing all experimental combinations were obtained and immediately subjected to DNA extraction and high-throughput sequencing. SOC and LTOC concentrations were determined by potassium dichromate oxidation followed by spectrophotometric measurement, whereas TN and TP were determined using the semi-micro Kjeldahl method and molybdenum–antimony colorimetry, respectively [39]. Soil pH was measured potentiometrically in a 1:5 soil-to-water suspension prepared with CO_2_-free deionized water [40].

High-throughput amplicon sequencing of soil microbial communities was performed by Shanghai Majorbio Bio-Pharm Technology Co., Ltd. (Shanghai, China). Genomic DNA was extracted from soil samples using the FastPure Soil DNA Isolation Kit (MJYH, Shanghai, China) according to the manufacturer’s instructions. The V3–V4 region of the bacterial 16S rRNA gene was amplified using primers 338F (5′-ACTCCTACGGGAGGCAGCAG-3′) and 806R (5′-GGACTACHVGGGTWTCTAAT-3′), whereas the fungal internal transcribed spacer 1 (ITS1) region was amplified using primers ITS1F (5′-CTTGGTCATTTAGAGGAAGTAA-3′) and ITS2R (5′-GCTGCGTTCTTCATCGATGC-3′). Purified amplicons were quantified, pooled in equimolar proportions, and subjected to paired-end sequencing using an Illumina MiSeq PE300 platform (Illumina, San Diego, CA, USA). Raw paired-end reads were quality-filtered using fastp v0.23.4 and subsequently merged using FLASH v1.2.11. Sequences were clustered into operational taxonomic units (OTUs) at 97% sequence similarity using UPARSE v11, with chimeric and non-target sequences removed before downstream analyses. Representative sequences of bacterial and fungal OTUs were taxonomically classified using RDP Classifier v2.11 at a confidence threshold of 0.70 against the SILVA Release 138.2 and UNITE Release 9.0 databases, respectively. Bacterial and fungal datasets were rarefied separately to the minimum sequencing depth within each dataset. The resulting normalized OTU abundance tables were used to calculate α-diversity indices and for taxonomic profiling, redundancy analysis (RDA), and co-occurrence network analysis.

### 2.5. Statistical and Modeling Analyses

Statistical analyses and data visualization were performed using R (v4.3.2), SPSS (v32.0.0; IBM Corp., Armonk, NY, USA), Origin 2024 (v10.1; OriginLab Corp., Northampton, MA, USA), and Gephi (v0.10). Litter decomposition rate and temperature sensitivity were characterized by *k* and *Q*_10_, respectively, whereas C, N, and P dynamics in the remaining litter were quantified using the NAI. Differences in *k* were assessed using two-way analysis of variance (ANOVA), with litter type (L), incubation temperature (T), and their interaction (L × T) included as fixed effects. Tukey’s honestly significant difference (HSD) test was subsequently used for multiple comparisons. *Q*_10_ values were compared among litter treatments separately for the 15–25 °C and 25–35 °C temperature intervals. Initial litter nutrient stoichiometry was characterized by C/N, C/P, and N/P ratios. Differences in these ratios among litter treatments were assessed using one-way ANOVA followed by Tukey’s HSD test. Pearson correlation analysis was used to characterize the overall associations of decomposition constants with incubation temperature and initial litter C/N, C/P, and N/P ratios, providing complementary evidence for the statistical associations between thermal conditions, initial litter stoichiometry, and decomposition dynamics. Linear models were further constructed to test the interactive effects of incubation temperature and initial litter stoichiometry on decomposition constants. When significant temperature × stoichiometry interactions were detected, temperature-specific slopes were examined to determine the direction and magnitude of the temperature-dependent variation in the relationship between initial litter stoichiometry and decomposition rates. Three-way ANOVA was used to assess the main and interactive effects of decomposition time, litter type, and incubation temperature on SOC, TN, TP, and pH.

Based on the rarefied OTU abundance table, bacterial and fungal α-diversity indices (Chao1, ACE, Shannon, and Simpson) were calculated using the “vegan” and “linkET” packages in R (v4.3.2). Taxonomic composition was profiled at the phylum level to evaluate relative abundance shifts under varying temperatures, incubation periods, and litter treatments. RDA evaluated the relationships between soil physicochemical properties, remaining litter mass, nutrient (C, N, and P) release rates, and microbial community structures across decomposition phases, revealing community-level environmental filtering. Co-occurrence network analysis was used to characterize statistical associations among microbial taxa under different incubation temperatures. Bacterial and fungal co-occurrence networks were constructed separately for each incubation temperature using OTUs with relative abundances > 0.01%. Pairwise Spearman rank correlations were calculated among the retained OTUs, and the resulting *p* values were adjusted for multiple comparisons using the Benjamini–Hochberg false discovery rate (FDR) procedure. Only robust and significant associations (|ρ| ≥ 0.70 and FDR-adjusted *p* < 0.05) were retained as network edges. Positive and negative associations were distinguished, and network topological properties, including the number of nodes, number of edges, average degree, clustering coefficient, and proportion of positive edges, were calculated and visualized using Gephi (v0.10).

PLS-SEM was applied as an exploratory multivariate approach to evaluate the relative contributions of experimental factors and ecological processes to four composite ecological constructs [41]. Predictors included incubation temperature, decomposition time, soil pH, and two dummy-coded litter contrasts (*C. lasiocarpa* vs. *C. angustifolia* and mixed litter vs. *C. angustifolia*), with *C. angustifolia* as the reference. Mode B composites were constructed from bacterial and fungal α-diversity, C- and N-related functional-module scores, *k* and C, N, and P nutrient accumulation indices, and SOC, TN, and TP, representing microbial α-diversity, C–N functional attributes, litter decomposition–nutrient dynamics, and soil C–N–P status, respectively. The model was fitted using 27 treatment-level observations, and significance was assessed using 5000 bootstrap resamples.

## 3. Results

### 3.1. Warming Effects on Litter Decomposition

Litter decomposition differed significantly among temperature and litter treatments (Table 1). *k* increased with temperature across all litter treatments and was significantly affected by litter treatment (*F*_2,18_ = 23.806, *p* < 0.001), incubation temperature (*F*_2,18_ = 223.422, *p* < 0.001), and their interaction (*F*_4,18_ = 14.922, *p* < 0.001). The highest *k* was observed for mixed litter at 35 °C (3.00 ± 0.24 yr^−1^), whereas the lowest was observed for *C. lasiocarpa* litter at 15 °C (1.01 ± 0.06 yr^−1^).

Temperature sensitivity also varied significantly among litter treatments. For *C. angustifolia*, *C. lasiocarpa*, and mixed litter, *Q*_10_ values were 1.28 ± 0.05, 1.85 ± 0.23, and 1.12 ± 0.14, respectively, over 15–25 °C, and 1.21 ± 0.01, 1.25 ± 0.09, and 1.71 ± 0.10, respectively, over 25–35 °C. Litter treatment significantly affected *Q*_10_ over both temperature intervals (15–25 °C: *F*_2,6_ = 17.205, *p* = 0.003; 25–35 °C: *F*_2,6_ = 41.240, *p* < 0.001). *Q*_10_ was highest for *C. lasiocarpa* over 15–25 °C and for mixed litter over 25–35 °C.

Pearson correlation analysis showed that temperature was positively correlated with *k* and stage-specific decomposition constants (*k*_1_ and *k*_2_) (Table 2). In contrast, correlations between *k* and initial litter C/N, C/P, and N/P ratios were weak.

**Figure 2:**
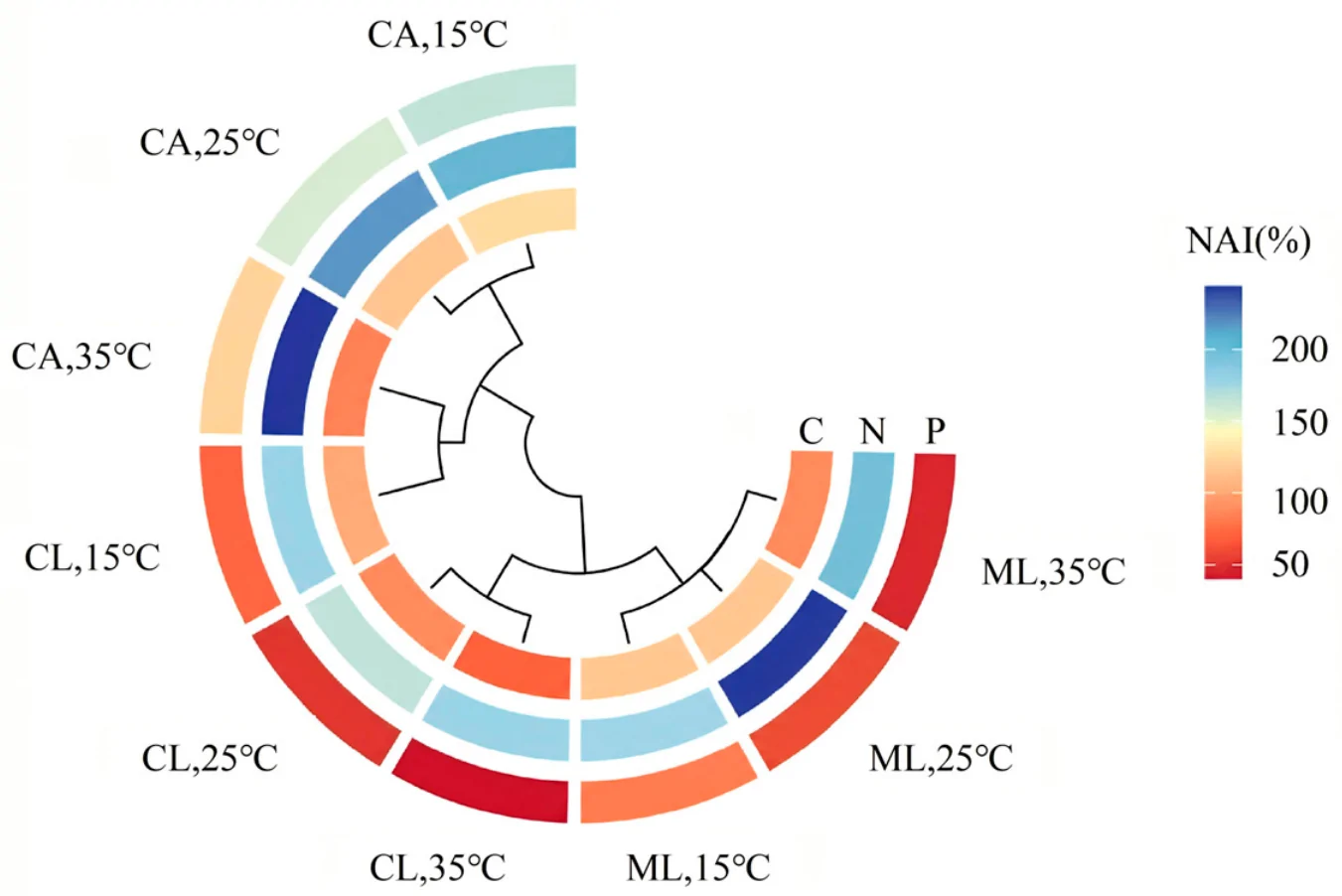
Cumulative nutrient accumulation indices (NAI) over the 90-d litter decomposition period. NAI values on day 90 represent the cumulative net nutrient balance relative to the initial litter nutrient pool over the 0–90 d decomposition period. Values > 100% indicate net accumulation, whereas values < 100% indicate net release. The sectors arranged around the circumference represent nine treatment combinations comprising three litter treatments—CA, *C. angustifolia*; CL, *C. lasiocarpa*; and ML, mixed litter—incubated at 15, 25, and 35 °C. The radially arranged variables represent the nutrient accumulation indices for carbon (NAI-C), nitrogen (NAI-N), and phosphorus (NAI-P). The color gradient from red (lower values) to blue (higher values) indicates increasing NAI values across treatment combinations. The central dendrogram shows the agglomerative hierarchical clustering of the nine treatment combinations based on NAI-C, NAI-N, and NAI-P. The three NAI variables were standardized using z-scores, Euclidean distances were calculated among treatment combinations, and hierarchical clustering was performed using Ward’s minimum-variance method (Ward.D2).

Litter C, N, and P accumulation indices differed among litter types and temperature treatments (Figure 2). The carbon accumulation index was below 100% in several treatments, indicating net C release, particularly under *C. lasiocarpa* at 35 °C. The nitrogen accumulation index remained above 100% across treatments, indicating N accumulation during decomposition. The phosphorus accumulation index showed treatment-dependent variation, with lower values in *C. lasiocarpa* and mixed litter at 35 °C.

### 3.2. S Oil C, N, and P Transformations During Litter Decomposition

**Figure 3:**
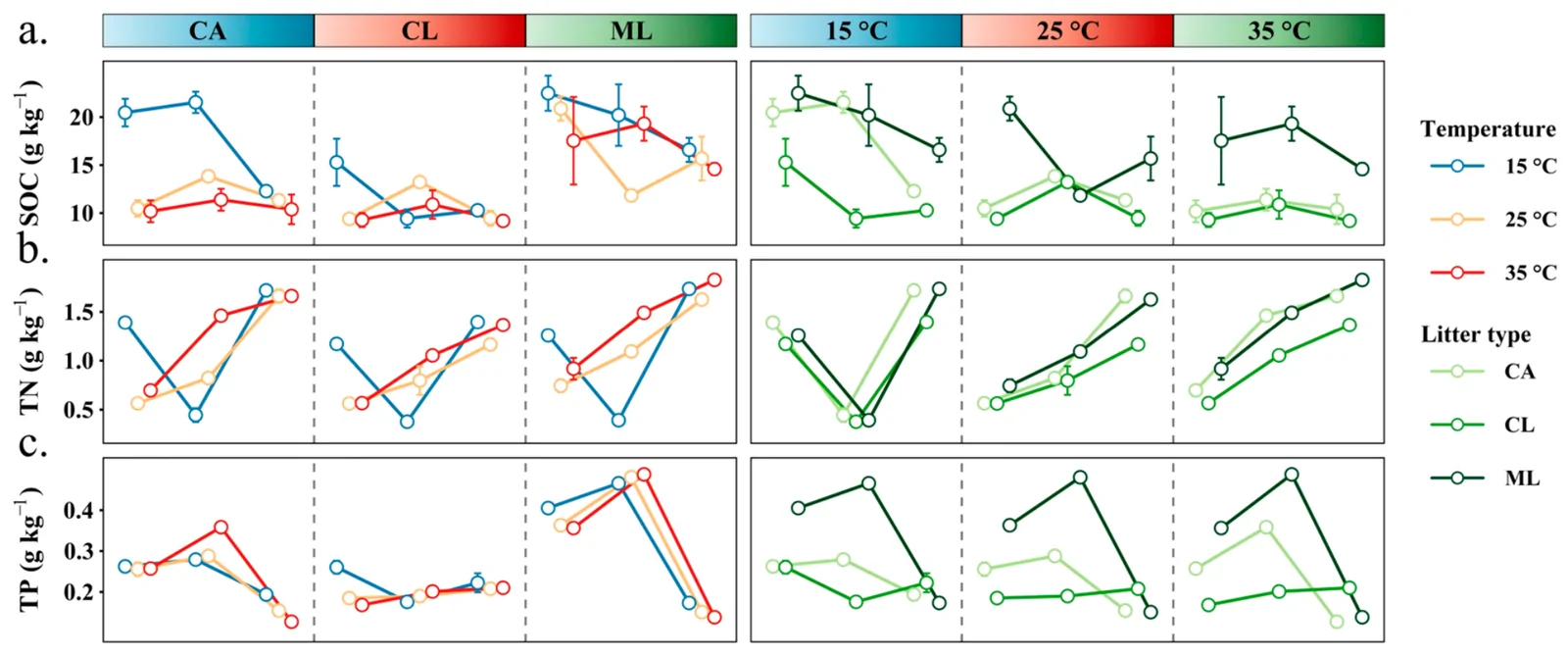
Changes in soil organic carbon (SOC), total nitrogen (TN), and total phosphorus (TP) during litter decomposition. Temporal variations in soil organic carbon (SOC; (**a**)), total nitrogen (TN; (**b**)), and total phosphorus (TP; (**c**)) during litter decomposition under different litter treatments and incubation temperatures (15, 25, and 35 °C). CA, CL, and ML denote *C. angustifolia* litter, *C. lasiocarpa* litter, and a mixture of the two litter species, respectively. Values represent means ± SE (*n* = 3). Solid lines connect the mean values across decomposition times.

Soil SOC, TN, and TP varied with decomposition time, litter type, and temperature (Figure 3). At day 20, SOC was lower under warming treatments than under 15 °C in single-litter treatments. At day 50, TN increased under 35 °C across litter treatments, and TP reached relatively high values under *C. angustifolia* and mixed litter. By day 90, SOC decreased across treatments, while TN and TP showed litter- and temperature-specific patterns.

### 3.3. Microbial Community Responses to Warming and Litter Decomposition

**Figure 4:**
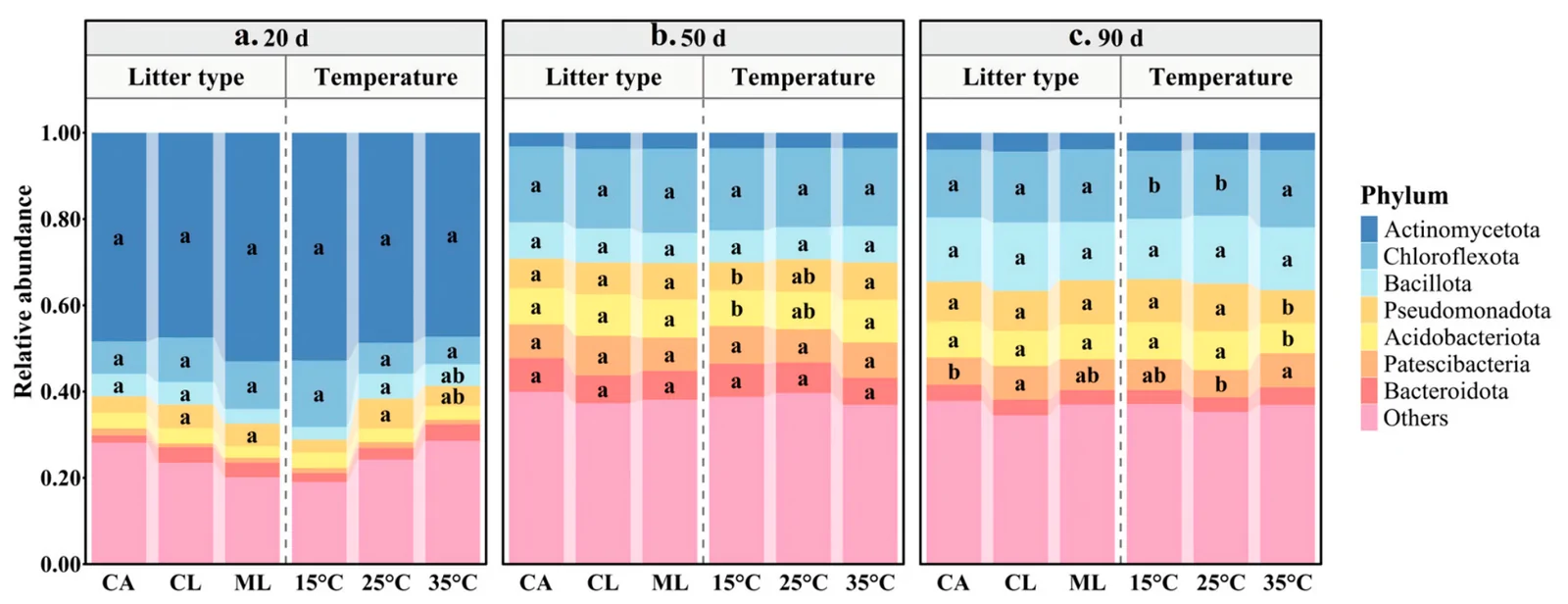
Relative abundances of dominant bacterial phyla during litter decomposition under different litter types and incubation temperatures. Phylum-level composition of bacterial communities after 20 (**a**), 50 (**b**), and 90 days (**c**) of decomposition. Bacterial community profiles were obtained from 27 composite soil samples, with nine profiles at each sampling time. Within each panel, the left section shows the bacterial community composition under different litter treatments, whereas the right section shows the composition at different incubation temperatures. CA, CL, and ML denote *C. angustifolia* litter, *C. lasiocarpa* litter, and a mixture of the two litter species, respectively. The incubation temperatures were 15, 25, and 35 °C. Taxa not displayed individually were grouped as “Others”. For each decomposition time and bacterial phylum, differences among litter treatments and among incubation temperatures were evaluated separately using one-way ANOVA followed by Tukey’s HSD test (*p* < 0.05). Different lowercase letters indicate significant pairwise differences within the corresponding litter-treatment or temperature comparison; groups sharing at least one letter are not significantly different.

The bacterial community was dominated by Actinomycetota, Chloroflexota, Bacillota, Pseudomonadota, and Acidobacteriota (Figure 4). Bacterial community composition exhibited stronger temporal shifts than temperature-driven variation during decomposition. Actinomycetota showed high relative abundance at day 20 and declined thereafter, whereas Chloroflexota, Bacillota, Pseudomonadota, and Acidobacteriota increased during the middle and late stages of incubation.

**Figure 5:**
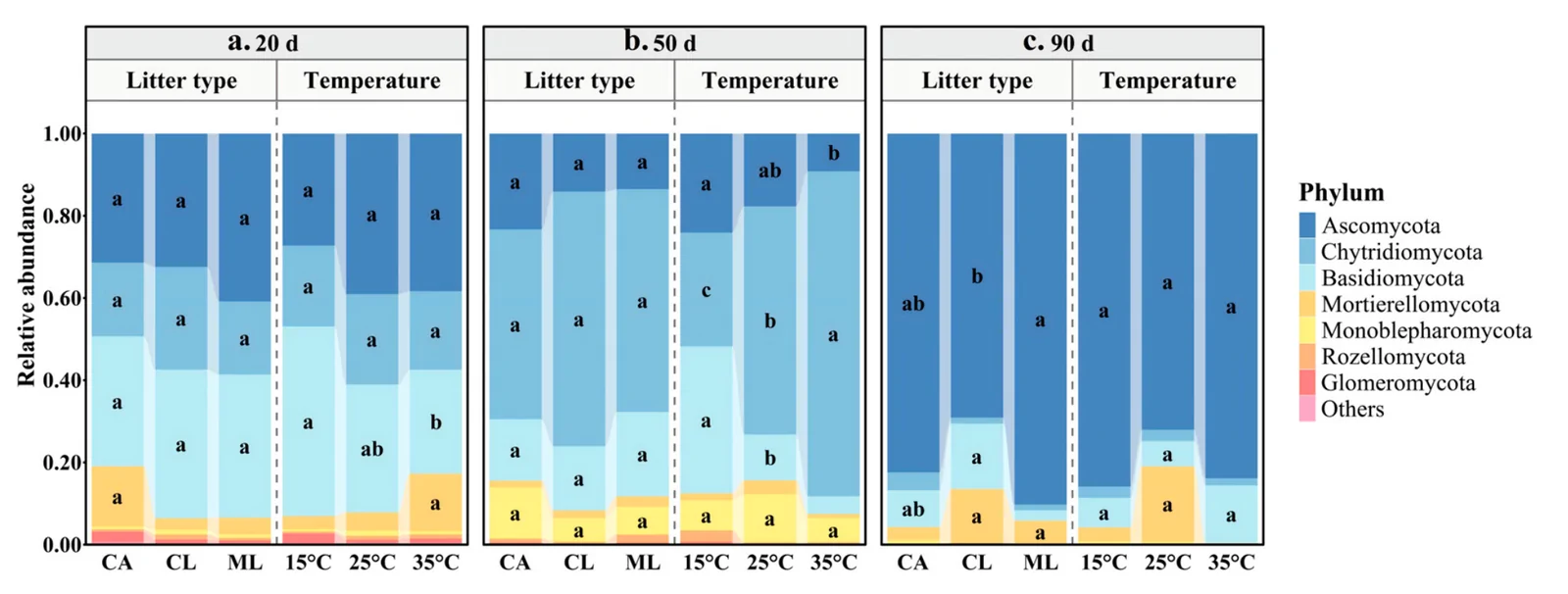
Relative abundances of dominant fungal phyla during litter decomposition under different litter types and incubation temperatures. Phylum-level composition of fungal communities after 20 (**a**), 50 (**b**), and 90 days (**c**) of decomposition. Fungal community profiles were obtained from 27 composite soil samples, with nine profiles at each sampling time. Within each panel, the left section shows fungal community composition under different litter treatments, whereas the right section shows fungal community composition at different incubation temperatures. CA, CL, and ML denote *C. angustifolia* litter, *C. lasiocarpa* litter, and a mixture of the two litter species, respectively. The incubation temperatures were 15, 25, and 35 °C. Fungal taxa not displayed individually were grouped as “Others.” For each decomposition time and fungal phylum, differences among litter treatments and among incubation temperatures were evaluated separately using one-way ANOVA followed by Tukey’s HSD test (*p* < 0.05). Different lowercase letters indicate significant pairwise differences within the corresponding litter-treatment or temperature comparison; groups sharing at least one letter are not significantly different.

The fungal community was mainly composed of Ascomycota, Chytridiomycota, Basidiomycota, and Mortierellomycota (Figure 5). Unlike bacterial communities, fungal assemblages exhibited pronounced sensitivity to temperature variation during decomposition. Ascomycota became more dominant at 35 °C and during the late decomposition stage, whereas Chytridiomycota and Basidiomycota showed higher relative abundance under 15 and 25 °C treatments.

**Figure 6:**
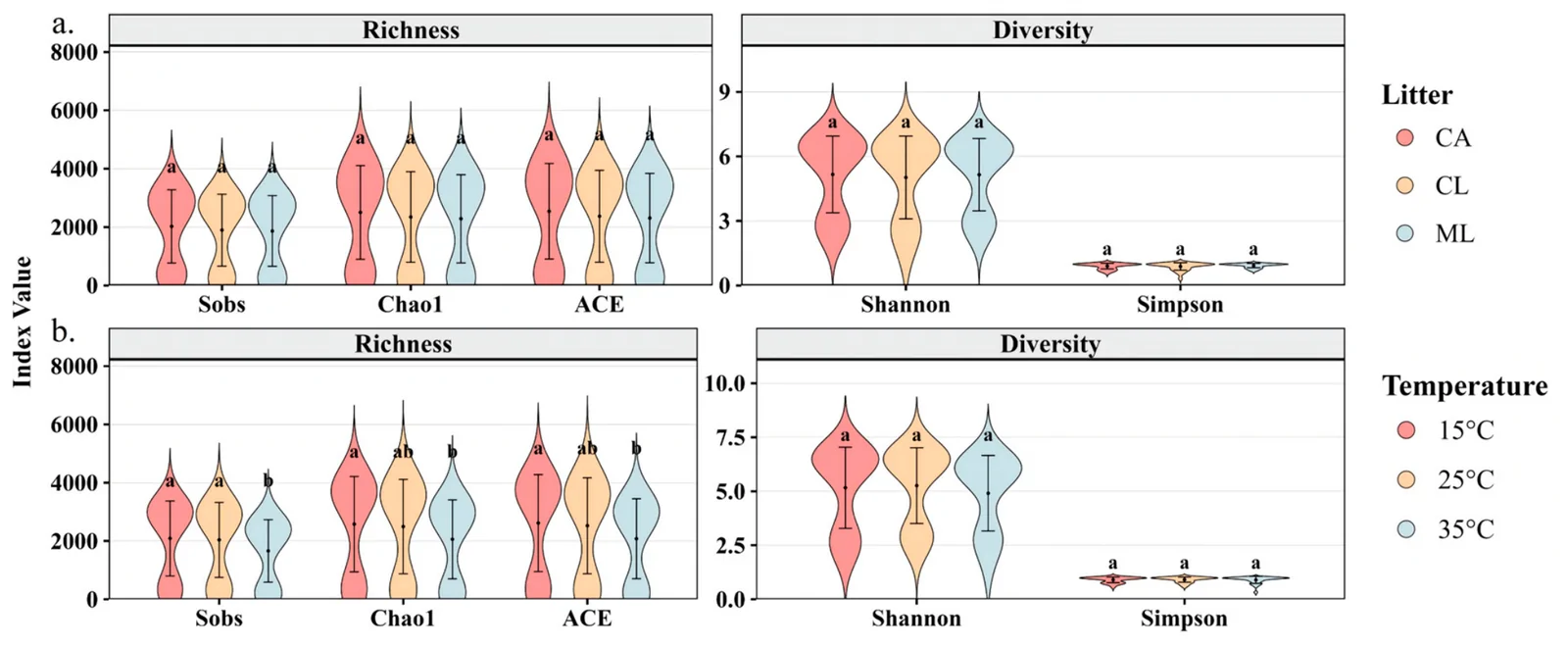
α-diversity indices of the bacterial community under different litter treatments. Violin plots of bacterial α-diversity indices illustrate (**a**) differences among litter configurations (CA, *C. angustifolia* litter; CL, *C. lasiocarpa* litter; and ML, mixed-litter), (**b**) incubation temperatures. All α-diversity indices were calculated separately for the 27 composite soil samples, with nine community profiles in each litter group. The width of each violin represents the probability density of the observations, black points and error bars represent means ± SD, respectively. Within each α-diversity index, different lowercase letters above the violins indicate significant differences among litter treatments or incubation temperatures at *p* < 0.05.

**Figure 7:**
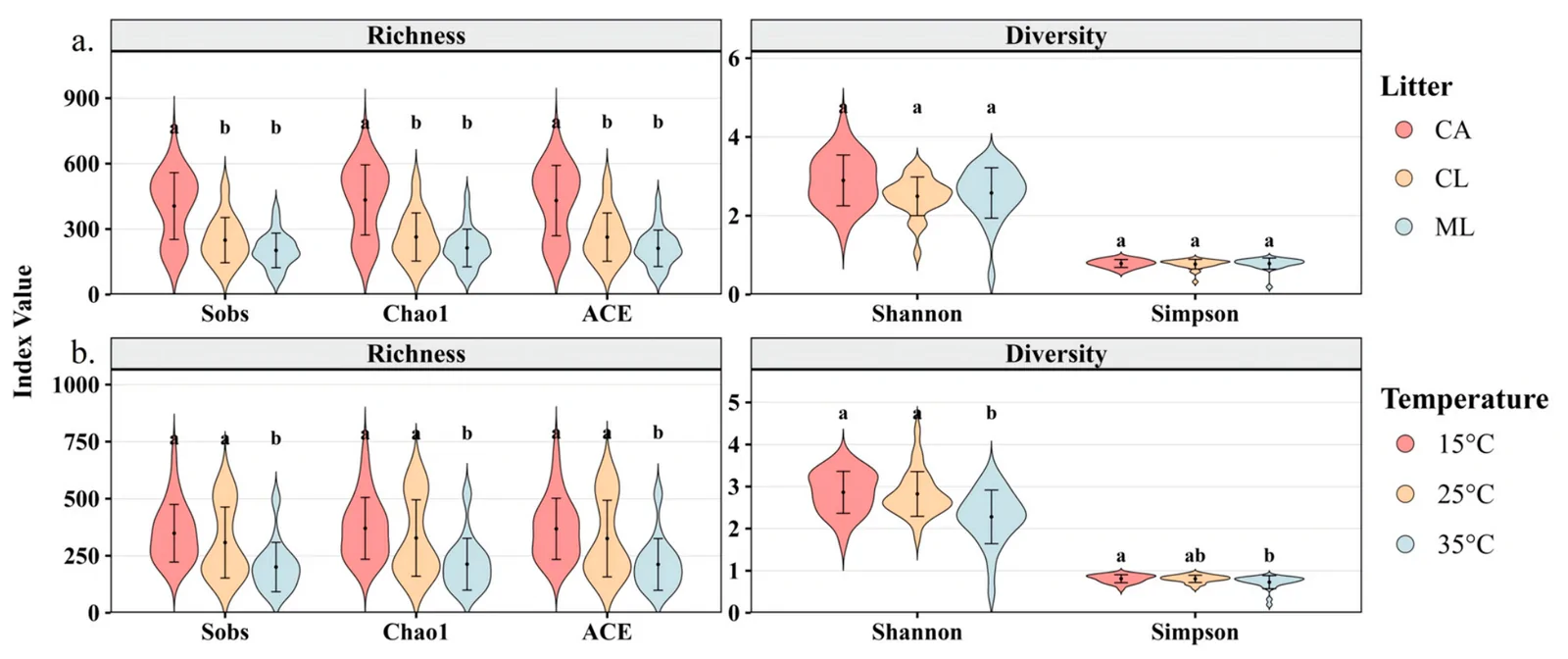
α-diversity indices of the fungal community under different litter treatments. Violin plots of fungal α-diversity indices illustrate (**a**) differences among litter configurations (CA, *C. angustifolia* litter; CL, *C. lasiocarpa* litter; and ML, mixed-litter), (**b**) incubation temperatures. All α-diversity indices were calculated separately for the 27 composite soil samples, with nine community profiles in each litter group. The width of each violin reflects the probability density of the observations, black points and error bars represent means ± SD, respectively. Within each α-diversity index, different lowercase letters above the violins indicate significant differences among litter treatments or incubation temperatures at *p* < 0.05.

Bacterial α-diversity showed limited variation among litter treatments, whereas incubation temperature exerted a stronger influence on bacterial richness than on diversity indices (Figure 6). Over the decomposition period, bacterial richness increased progressively from 20 to 90 d, whereas diversity indices increased from 20 to 50 d and remained relatively stable thereafter (Figure S1). Fungal α-diversity exhibited stronger temperature sensitivity than bacterial α-diversity during litter decomposition (Figure 7). Fungal richness and diversity indices generally declined under 35 °C conditions, whereas *C. angustifolia* litter generally showed higher fungal α-diversity than *C. lasiocarpa* litter.

**Figure 8:**
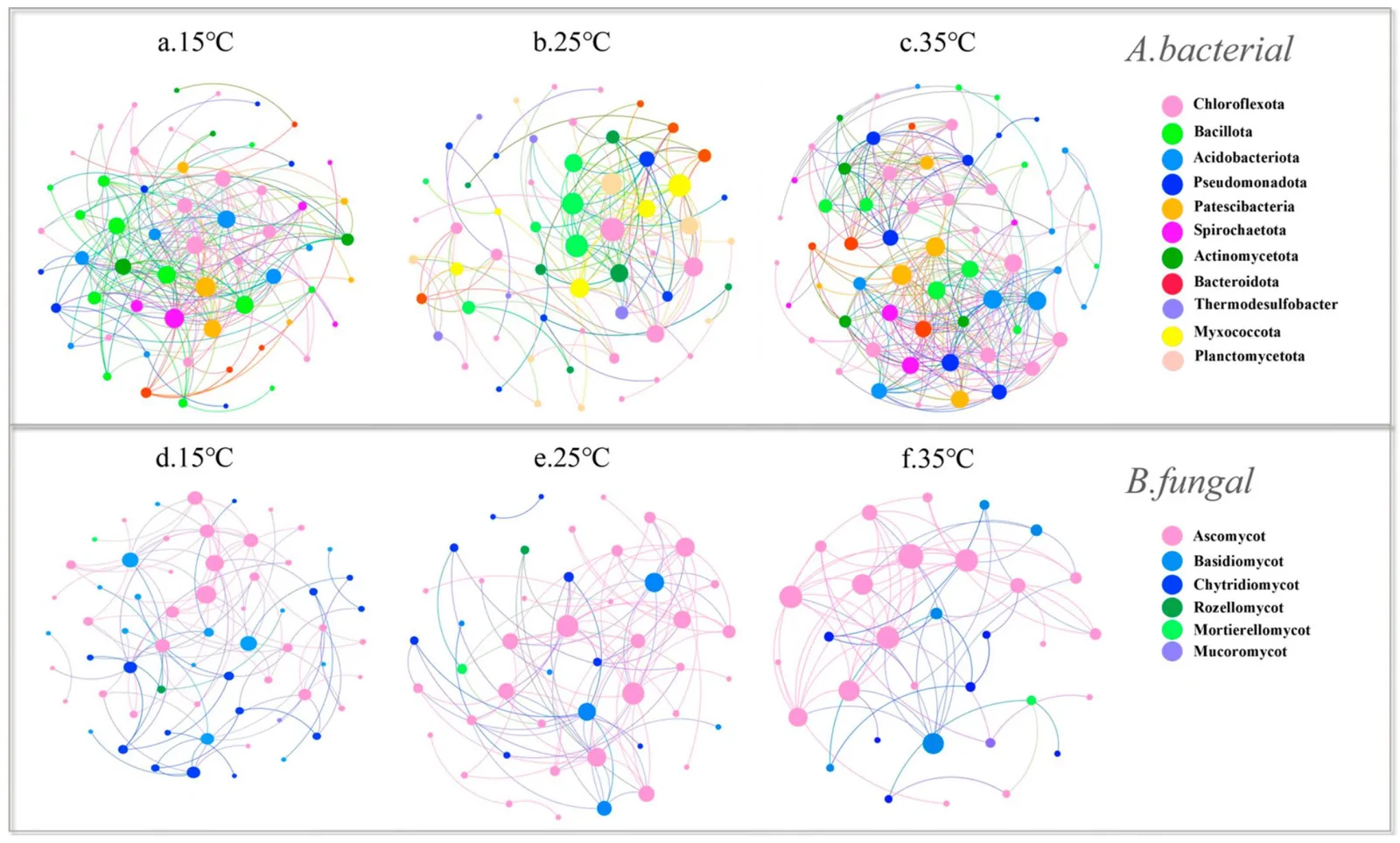
Co-occurrence networks of bacterial and fungal communities under different incubation temperatures. Panels (**a**–**c**) show bacterial networks at 15, 25, and 35 °C, respectively; panels (**d**–**f**) show fungal networks at the same respective temperatures. The 27 sequencing profiles represented all combinations of three litter treatments, three incubation temperatures, and three decomposition times. Accordingly, each temperature-specific network was constructed from nine profiles representing the three litter treatments at 20, 50, and 90 d. Each node represents an OTU; node color indicates phylum-level taxonomic affiliation, and node size is proportional to degree. Edges represent Spearman correlations with |ρ| ≥ 0.90 and false discovery rate (FDR)-adjusted *p* < 0.05. Within each temperature group, OTUs occurring in at least 60% of samples were retained, and the 200 most abundant OTUs were selected for correlation analysis prior to network construction. For visualization, only OTUs assigned to the eight most represented classified phyla were retained; unclassified or norank taxa and isolated nodes were excluded.

Microbial co-occurrence networks varied across temperature treatments (Figure 8; Table 3 and Table 4). For bacterial communities, increasing temperature enhanced network connectivity, with edges increasing from 204 at 15 °C to 300 at 35 °C and average degree increasing from 6.80 to 9.38. For fungi, the number of nodes decreased from 63 at 15 °C to 32 at 35 °C, while the average degree and clustering coefficient increased from 3.08 and 0.38 to 4.44 and 0.66, respectively.

### 3.4. Linkages Among Litter Decomposition, Microbial Reorganization, and Soil Nutrient Transformation

**Figure 9:**
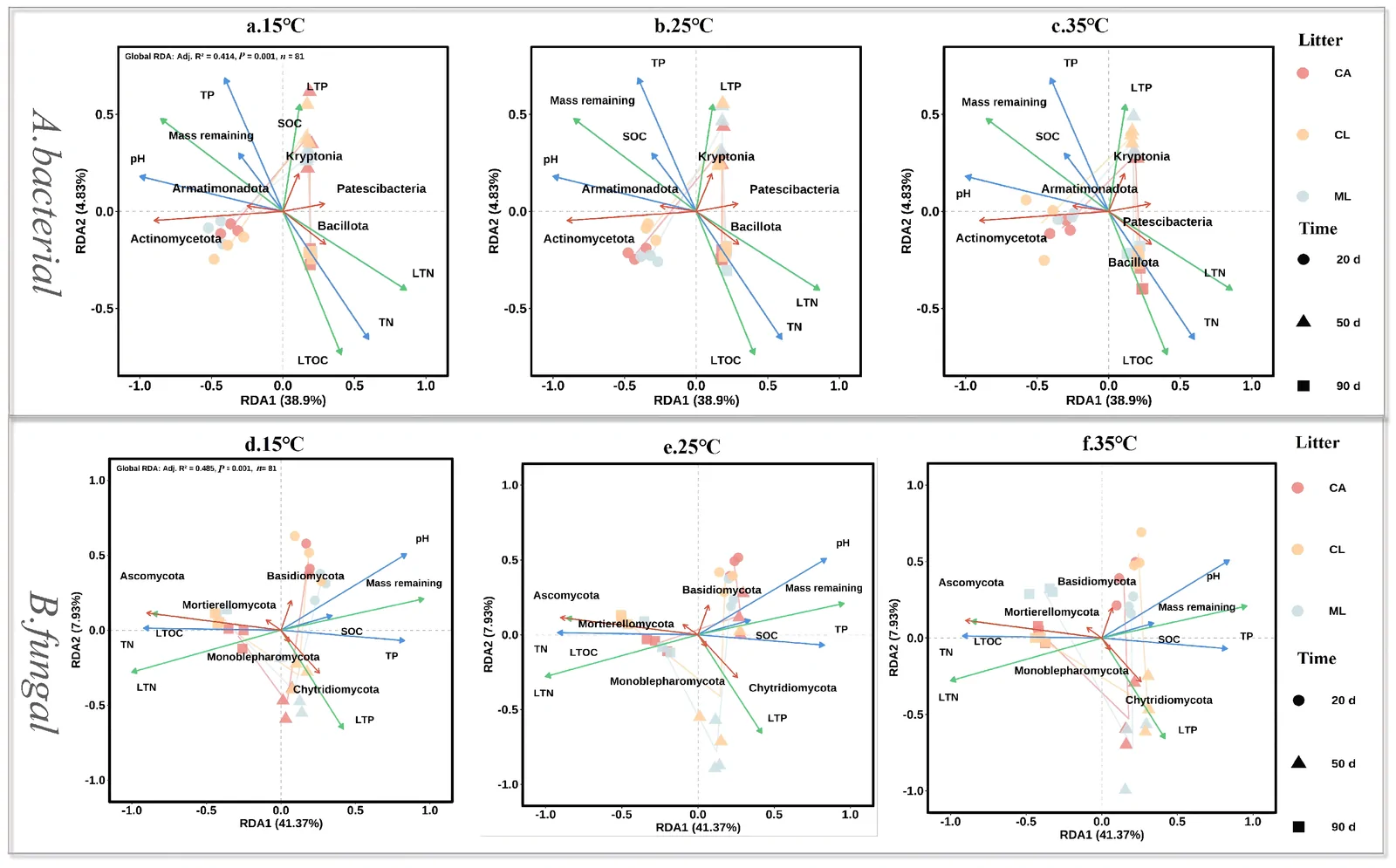
Redundancy analysis (RDA) of microbial community structure. RDA ordination plots show bacterial (A, (**a**–**c**)) and fungal (B, (**d**–**f**)) communities at 15 °C (**a**,**d**), 25 °C (**b**,**e**), and 35 °C (**c**,**f**). The bacterial and fungal datasets each contained community profiles from 27 composite soil samples. For RDA, soil and litter measurements from the 81 microcosms were averaged within each litter type × incubation temperature × harvest-time combination. These means were then matched to the 27 corresponding community profiles. Point colors indicate litter treatments (CA, *C. angustifolia*; CL, *C. lasiocarpa*; ML, mixed litter), and point shapes indicate decomposition times (20, 50, and 90 d). Blue and green arrows represent soil variables (SOC, TN, TP, and pH) and litter variables (mass remaining, LTOC, LTN, and LTP), respectively. Red arrows represent the five bacterial or fungal phyla with the longest RDA vectors. The global bacterial and fungal RDA models had adjusted *R*^2^ values of 0.414 and 0.485, respectively (both *p* = 0.001).

Redundancy analysis revealed significant associations between bacterial and fungal community composition and soil and litter properties during decomposition (Figure 9). The global RDA models were significant for both bacterial (adjusted *R*^2^ = 0.414, *p* = 0.001) and fungal communities (adjusted *R*^2^ = 0.485, *p* = 0.001). Bacterial community composition was associated with gradients in SOC, TN, TP, and soil pH, as well as litter mass remaining, LTOC, LTN, and LTP, with distinct sample distributions across incubation temperatures and decomposition times. Fungal communities exhibited a distinct ordination pattern and were similarly associated with both soil and litter properties across incubation temperatures and decomposition times.

### 3.5. Drivers of Soil Nutrient Transformations During Litter Decomposition

Three-way analysis of variance (ANOVA) demonstrated that incubation time (D), litter type (L), and temperature (T) significantly affected SOC, TN, TP, and pH during litter decomposition (Table 5). All three factors exerted highly significant effects across the measured soil properties (*p* < 0.001). Specifically, L exerted a stronger influence on SOC, whereas D was the primary driver of variations in TN, TP, and pH. Furthermore, soil properties were subject to multidimensional interactive regulation, as shown by the significant two-way interactions of D × T and L × T on SOC, TN, TP, and pH. The three-way interaction (D × L × T) also significantly modulated all soil parameters, further demonstrating the highly coupled regulation of soil biogeochemical properties during litter decomposition.

**Figure 10:**
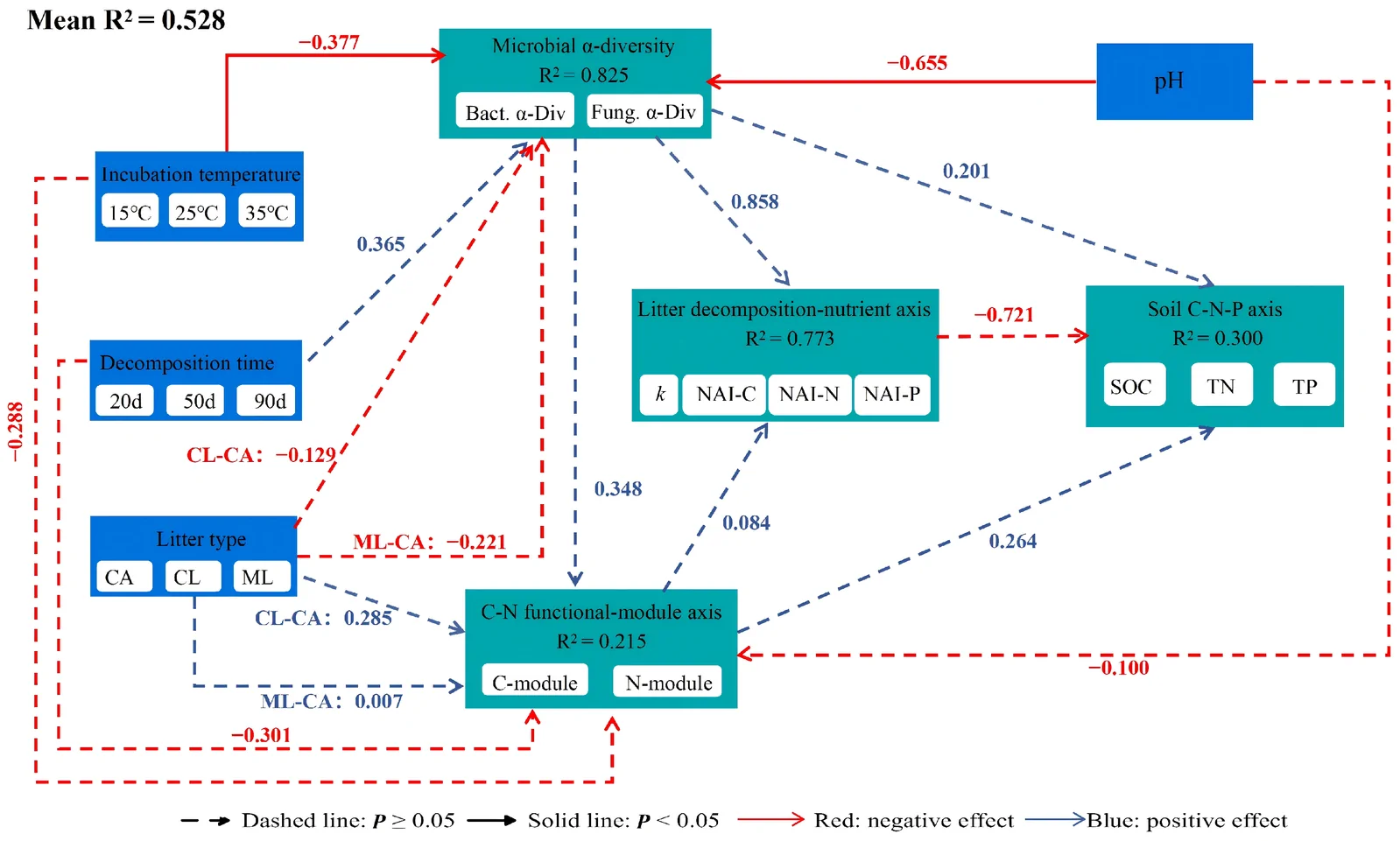
Partial Least Squares Structural Equation Model (PLS-SEM) analysis of linkages among warming, litter decomposition, microbial responses, and soil nutrient dynamics. The PLS-SEM illustrates the direct structural pathways linking incubation temperature, decomposition time, litter type, soil pH, microbial α-diversity, the C–N functional-module axis, the litter decomposition–nutrient axis, and the soil C–N–P axis. The model was fitted using treatment-level observations (*n* = 27). Each observation represented one combination of litter treatment, incubation temperature (15, 25, or 35 °C), and harvest time (20, 50, or 90 d). replicates per experimental combination. The experiment comprised 81 microcosms, with three replicates per combination. Within each combination, soil properties and litter nutrient accumulation indices were averaged across the three microcosms. For microbial sequencing, soil subsamples from the three microcosms within each combination were pooled before DNA extraction to form one composite sample. Microbial variables derived from the 27 composite samples were then matched to the corresponding soil and litter means by experimental combination to construct the PLS-SEM dataset. Litter treatments comprised *C. angustifolia* litter (CA), *C. lasiocarpa* litter (CL), and mixed litter (ML). CA served as the reference category; thus, CL–CA and ML–CA denote the contrasts of CL and ML, respectively, relative to CA. Microbial α-diversity was modeled as a Mode B composite comprising bacterial and fungal α-diversity. The C–N functional-module axis was modeled as a Mode B composite comprising the carbon-related (C-module) and nitrogen-related (N-module) functional modules. The litter decomposition–nutrient axis comprised *k* and the nutrient accumulation indices of carbon (NAI-C), nitrogen (NAI-N), and phosphorus (NAI-P), whereas the soil C–N–P axis comprised SOC, TN, and TP. Numbers adjacent to the arrows represent standardized direct path coefficients. The statistical significance of direct paths was assessed using two-sided bootstrap tests based on 5000 resamples. Blue and red arrows indicate positive and negative effects, respectively, whereas solid and dashed arrows denote significant (*p* < 0.05) and nonsignificant (*p* ≥ 0.05) paths, respectively. The R^2^ values within the boxes indicate the proportions of variance explained in the endogenous constructs. Mean R^2^ represents the arithmetic mean of the R^2^ values across the four endogenous constructs.

**Figure 11:**
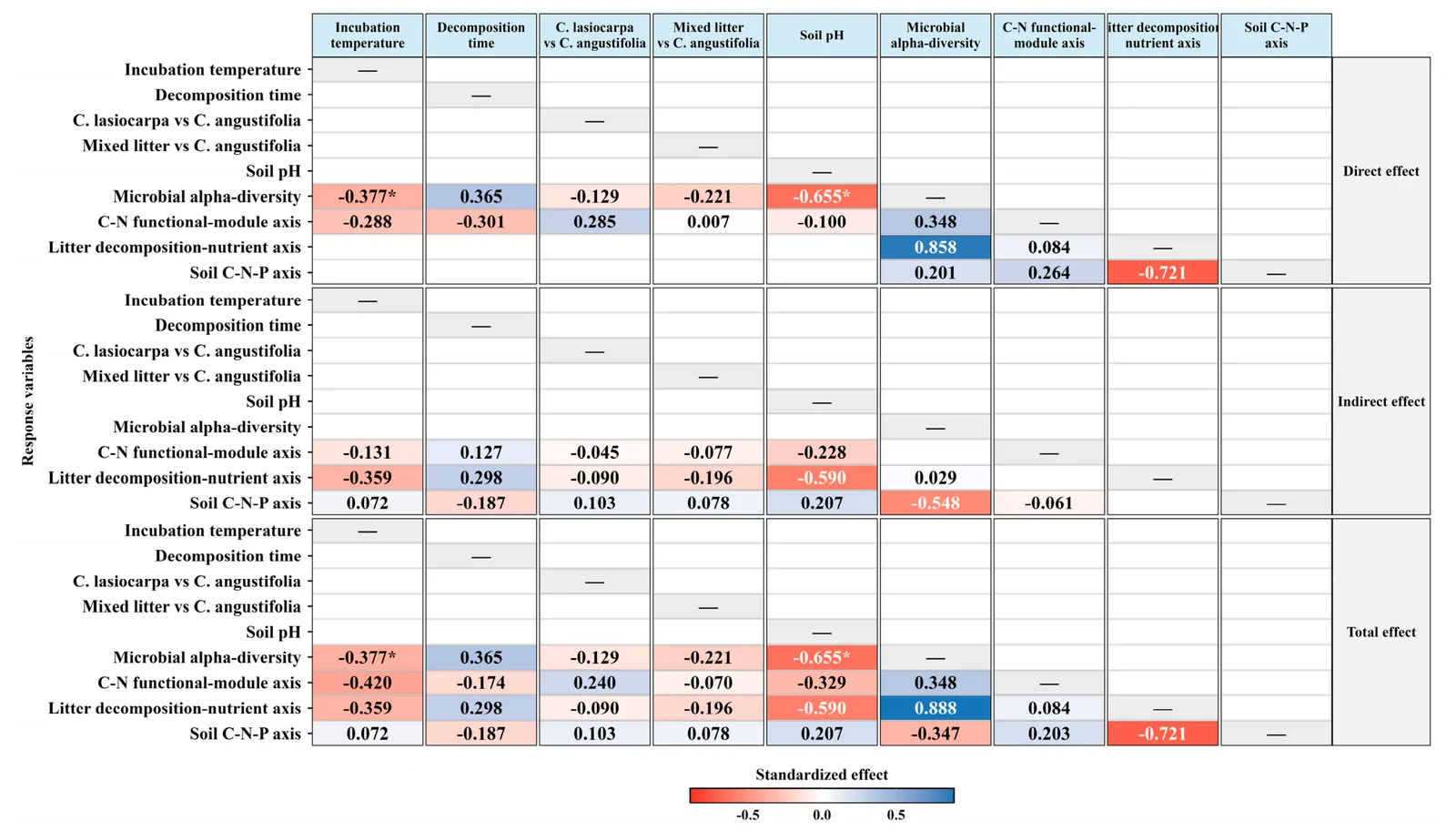
Direct, indirect, and total effects in the partial least squares structural equation model (PLS-SEM). The heatmap shows the standardized direct, indirect, and total effects estimated using the same PLS-SEM presented in Figure 10. The analysis was based on 27 treatment-level observations (n = 27). Statistical significance was evaluated using two-sided bootstrap tests with 5000 resamples. Asterisks indicate significance levels: * *p* < 0.05. Positive and negative standardized effects are represented by blue and red shading, respectively, with color intensity indicating effect magnitude. *C. angustifolia* litter (CA) was used as the reference category for litter type; therefore, “*C. lasiocarpa* vs. *C. angustifolia*” and “Mixed litter vs. *C. angustifolia*” represent the contrasts of CL and ML relative to CA, respectively. Columns represent predictor variables and rows represent response variables. Blank cells indicate that no direct or indirect effect was specified or generated in that direction, whereas em dashes indicate the same variable or comparisons that were not applicable.

Following the predefined conceptual framework, PLS-SEM was applied to evaluate the direct, indirect, and total path relationships linking incubation temperature, decomposition time, litter treatment, and soil pH with microbial α-diversity, the C–N functional-module axis, the litter decomposition–nutrient axis, and the soil C–N–P axis (Figure 10). Incubation temperature and soil pH showed significant negative direct associations with microbial α-diversity, with standardized path coefficients of −0.377 (*p* = 0.038) and −0.655 (*p* = 0.023), respectively (Figure 11). In contrast, neither decomposition time nor the litter-treatment contrasts had significant direct effects on microbial α-diversity. No significant indirect effects were detected, and none of the total effects on the C–N functional-module, litter decomposition–nutrient, or soil C–N–P axes reached statistical significance (Figure S3).

## 4. Discussion

### 4.1. Warming Accelerates Litter Decomposition and Drives Asynchronous, Stage-Specific Release of Carbon, Nitrogen, and Phosphorus

Simulated warming significantly accelerated litter mass loss and increased *k* across varying substrate configurations, with *k* peaking at 35 °C and reaching its minimum at 15 °C (Table 1). This pattern supports the hypothesis that warming accelerates wetland litter decay, aligning with the global consensus of enhanced decay rates under climate warming [42]. Temperature is a dominant physical factor governing biochemical reaction kinetics and microbial metabolic activities. Specifically, elevated temperatures increase the proportion of reactant molecules with sufficient energy to overcome the activation-energy barrier, thereby increasing reaction rates [43]. This kinetic effect may enhance microbial metabolic activity and extracellular enzyme-mediated degradation processes, thereby facilitating the mineralization of organic litter fractions. Concurrently, the releases of C, N, and P were highly decoupled, exhibiting pronounced temporal phases along the decomposition continuum. Specifically, the early phase was dominated by the rapid mass loss of labile carbon fractions, whereas the release and exchange of N and P with the soil nutrient pool gradually intensified during the mid-to-late stages. These dynamics suggest that warming not only enhances overall mass decay rates but also alters the temporal stoichiometry and release rhythms of distinct elements.

Notably, the association between temperature and decomposition appeared more pronounced during the early phase than during the later phase. Specifically, both *k*_1_ and *k*_2_ were significantly positively correlated with temperature, although the correlation coefficient was numerically higher for *k*_1_ (r = 0.888) than for *k*_2_ (r = 0.746) (Table 2). This patterned response is consistent with classic multi-stage decay kinetics, where the initial phase is characterized by a high abundance of labile, non-crystalline carbohydrates (e.g., soluble sugars and cellulose) [44]. These fractions possess high bioavailability and are sensitive to temperature fluctuations and microbially driven metabolic acceleration. As these labile fractions are depleted, the proportion of recalcitrant macromolecules (e.g., lignin and physically encapsulated complex organic compounds) increases in the remaining residues [45]. This shift, together with declining substrate accessibility and changes in extracellular enzyme activity and microbial physiology, may have contributed to the weaker apparent warming response during the later stage of decomposition. Concurrently, within the 25–35 °C interval, the mixed-litter treatment exhibited significantly greater temperature sensitivity (*Q*_10_ = 1.71 ± 0.10) than either single-species treatment and also showed the highest *k* at 35 °C. Mixing litter species provides high chemical heterogeneity. This diversity facilitates cross-substrate nutrient transfer, whereby soluble nutrients (e.g., nitrogen and phosphorus) released from high-quality litter migrate to and are utilized by microbial decomposers at the interface of adjacent low-quality litter [46]. This mechanism effectively alleviates localized nutrient limitations and stimulates cooperative mineralization among distinct microbial functional groups, ultimately triggering positive non-additive (synergistic) decomposition effects [47].

However, we did not observe a sustained, synchronous promotional effect of warming on C, N, and P release. Compared to the rapid loss of carbon fractions during the early phase, N and P release dynamics are governed by microbial immobilization, remineralization, and elemental exchange across the soil–litter interface, resulting in temporal and directional mismatches with C release [48]. From an ecological stoichiometry perspective, elemental turnover during decomposition is not modulated by temperature alone; instead, it is interactively regulated by microbial resource demands, residue stoichiometry, and the specific decay stage [49,50]. Therefore, the warming effects elucidated in this study are better conceptualized as a recalibration of decomposition progress and multi-elemental release rhythms, rather than a uniform acceleration of release rates for all nutrients. Because this experiment was conducted under constant temperature and moisture conditions, it cannot fully capture the impacts of natural wetland environmental fluctuations, such as diurnal temperature oscillations, water table fluctuations, and freeze–thaw cycles on element release dynamics.

### 4.2. Relationships Between Initial Litter Stoichiometry and Decomposition Under Constant High-Moisture Conditions

Across the two wetland plant species and their mixture, initial litter C/N, C/P, and N/P ratios showed weak overall correlations with *k* (Table 2). These relationships were observed during the 90-day incubation at 100% maximum water-holding capacity. This pattern describes the association between initial litter stoichiometry and decomposition within the litter treatments and environmental conditions examined. Associations between litter traits and decomposition may vary with the range of trait variation and the decomposition period examined [51,52]. The two species and their mixture included in this experiment represented a restricted range of litter characteristics. Under the constant high-moisture conditions of this experiment, nutrient exchange with the surrounding soil may also have influenced the resources available to decomposers.

The litterbags were embedded in wetland soil maintained at 100% maximum water-holding capacity, allowing close contact between the litter and the surrounding soil matrix. The exchange of dissolved nutrients and organic compounds across the soil–litter interface may have supplemented litter-derived resources during decomposition [53]. Such exchange may help explain the weak association between initial litter stoichiometry and decomposition rates under the conditions examined. Microbial resource demands and substrate-use strategies change over time. Decomposition rates may therefore reflect the combined influences of initial substrate quality, external nutrient inputs, microbial community composition, and decomposition stage [54]. In this experiment, decomposition rates varied across incubation temperatures alongside changes in microbial community profiles. Initial litter stoichiometric ratios showed weak overall associations with *k*. These observations offer complementary perspectives on decomposition dynamics in the soil–litter system examined.

However, these findings do not imply that initial litter stoichiometry universally loses its predictive value for decomposition in wetland ecosystems. In contrast to their weak relationships with initial stoichiometric ratios, decomposition constants were positively correlated with incubation temperature (Table 2), consistent with our expectation that warming would accelerate litter decomposition. This pattern is consistent with growing evidence that the controls on litter decomposition are context dependent, arising from interactions among climate, litter traits, decomposer communities, and local environmental conditions. Under high-moisture wetland conditions, nutrient exchange between litter and the surrounding soil matrix may partially decouple initial litter stoichiometry from nutrient availability at the decomposition interface, thereby reducing its predictive value for decomposition rates [55]. Such soil–litter nutrient exchange may therefore partly explain the weak relationships between initial stoichiometric ratios and decomposition rates observed in our experiment. Importantly, our experiment included only two litter species and their mixture, and therefore encompassed a relatively narrow range of initial stoichiometric variation. Previous studies have shown that the apparent importance of litter traits can depend on the breadth of trait variation represented within a study [56]. The weak overall relationships observed here should be interpreted in the context of the limited range of litter traits and the 90-day incubation under constant high-moisture conditions. Including more species and mixtures, broader stoichiometric gradients, and longer decomposition periods in future comparisons would help assess the consistency of this pattern. Factorial experiments manipulating temperature and soil moisture could further clarify how these conditions influence the relationship between initial litter stoichiometry and decomposition.

### 4.3. Microbial Community Succession and Network Reorganization Link Litter Decomposition to Soil Nutrient Transformation

Composite community profiles varied over time during litter decomposition, coinciding with changes in soil nutrient status. Bacterial α-diversity was generally higher at later harvests. Community composition shifted from relatively high Actinomycetota abundance toward greater representation of Chloroflexota and Bacillota (Figure 4 and Figure 6). day 90 (Figure 5 and Figure 7). Fungal community profiles showed greater dominance of Ascomycota by day 90 (Figure 5 and Figure 7). This successional trajectory aligns with classic niche and microbial succession theories. Specifically, during early decomposition, the rapid release of labile carbon (such as soluble sugars) provides competitive advantages for copiotrophic, taxa generally associated with copiotrophic resource-use strategies, including members of Actinomycetota adapted to high-resource niches. As these labile fractions are depleted, the community transitions to oligotrophic, K-strategist decomposers (such as Chloroflexota and Ascomycota) possessing complex lignin- and cellulose-degrading enzyme systems [57]. RDA described associations between composite bacterial community profiles and the corresponding soil and litter variables aggregated by experimental combination (Figure 9). These patterns provide a basis for exploring covariation between community composition and nutrient status during decomposition. However, the analysis does not resolve relationships among individual microcosms within each experimental combination.

Co-occurrence networks constructed from composite community profiles differed among incubation conditions (Figure 8). At 35 °C, the bacterial network had the most edges. Compared with the fungal network at 15 °C, the network at 35 °C had fewer nodes but a higher average degree. At 35 °C, positive edges accounted for 97.67% of bacterial links and 92.96% of fungal links (Table 3 and Table 4). These proportions reflect predominantly positive covariation in the relative abundances of connected taxa across the sampled litter treatments and decomposition stages. Temperature-related changes in substrate turnover may alter resource availability and soil conditions for decomposer communities. Similar responses to these changes among taxa with shared environmental preferences may contribute to the positive associations observed during litter decomposition. At each incubation temperature, networks were constructed using community profiles spanning three litter treatments and three harvest times. Network topology thus summarizes associations among microbial abundances across contrasting substrate conditions and decomposition stages. Table 5 reports significant effects of incubation time, litter configuration, and temperature on SOC, TN, and TP, along with their interaction effects. The PLS-SEM results describe modeled associations among environmental conditions, microbial attributes, decomposition processes, and soil nutrient status (Figure 10 and Figure 11). Community profiles and co-occurrence networks describe microbial variation alongside changes in litter decomposition and soil nutrient status. These observations complement the RDA and PLS-SEM analyses by describing community patterns across experimental combinations. Variation in network connectivity and clustering summarizes associations among taxa, complementing the description of individual taxon abundance patterns. These network properties provide a basis for formulating hypotheses about shared microbial responses to changing conditions during litter decomposition [58].

Microbial α-diversity showed no consistent monotonic pattern across incubation conditions [59]. In the composite community profiles, dominant bacterial phyla showed relatively limited variation. Fungal richness and diversity were generally lower at 35 °C (Figure 7). The resident soil microbiome of the Xingkai Lake wetland is adapted to cold-temperate conditions, enabling bacteria to maintain functional stability through dormancy, metabolic regulation, and functional redundancy [60]. This capacity underpins the robust resilience of the bacterial community to thermal disturbances within a moderate temperature range. Conversely, fungal communities exhibited higher sensitivity to elevated temperatures. The 35 °C treatment exerted intense environmental filtering, causing psychrophilic or temperature-sensitive taxa to enter dormancy or decline precipitously [61,62]. Consequently, the fungal community converged toward heat-tolerant groups, such as dominant Ascomycota taxa [63]. This suggests a non-linear relationship between taxonomic diversity and ecological functioning; relying solely on taxonomic α-diversity indices, such as the Shannon index, may be insufficient to fully capture microbial regulatory capacity over multi-elemental biogeochemical turnover. Instead, community composition, functional guilds, and co-occurrence network topologies should be considered together.

The association patterns reported here were derived from relative-abundance data. Each temperature-specific network was constructed from nine composite community profiles spanning litter treatments and harvest times. Ecological interpretation of these associations should therefore account for the sampling structure and the variation in substrate conditions and decomposition stages represented in these profiles. In the experimental design, assigning a single incubator to each temperature treatment confounded temperature and incubator effects. Microcosm replicates characterized variation within the assigned incubation conditions, but independent incubator replication was unavailable. Accordingly, temperature-related differences should be interpreted within the specific incubation conditions examined. Static microcosms maintained at constant temperature and moisture provided a simplified representation of wetland conditions. In natural wetlands, water table fluctuations, freeze–thaw cycles, plant root activity, and soil fauna-mediated litter fragmentation may influence decomposition. Future experiments should include multiple independent incubators per temperature treatment, random allocation of temperature treatments to incubators, and separate sequencing of individual microcosm samples. Analyses should account for the nested experimental structure. Targeted co-culture experiments and stable-isotope tracing could help distinguish shared environmental responses from specific biological interactions and assess their relevance to nutrient transformations. Complementary long-term in situ warming experiments incorporating hydrological variability and multitrophic biological processes could help assess the relevance of these laboratory observations to natural wetlands. Together, these approaches could improve the assessment of microbial responses and their associations with litter decomposition and soil nutrient dynamics across environmental contexts.

## 5. Conclusions

The Xingkai Lake wetland represents a vital carbon sink. Focusing on dominant macrophytes, this study elucidated the interconnected mechanisms linking plant litter decomposition, microbial community responses, and soil nutrient transformations under simulated warming. The findings demonstrated that elevated temperatures significantly accelerated litter mass loss. Notably, mixed litter exhibited a pronounced synergistic decay response under moderate-to-high temperature regimes (25–35 °C), indicating that temperature was a major environmental regulator of decomposition kinetics under the experimental conditions. Composite microbial community profiles varied across incubation conditions and harvest times. Co-occurrence network topology differed among incubation conditions. These observations provided a microbial community context for the asynchronous C, N, and P dynamics recorded in soil and litter measurements. Soil nutrient transformations were associated with incubation temperature, litter treatment, incubation time, and their interactions. For the two wetland plant species and their mixture, initial litter C/N, C/P, and N/P ratios showed weak overall correlations with decomposition constants. These correlations were observed during the 90-day incubation at 100% maximum water-holding capacity. Across experimental combinations, microbial community profiles and co-occurrence patterns varied alongside litter decomposition and C–N–P dynamics. These microbial responses may contribute to nutrient release and immobilization patterns during decomposition, but their direct integrated effects on soil C–N–P status were not supported by the PLS-SEM results. This simulated warming experiment suggests that increasing temperature can accelerate litter decomposition and modify C, N, and P dynamics during the early stages of decomposition in the Xingkai Lake wetland. These findings highlight the potential implications of climate warming for nutrient cycling processes and carbon balance in high-latitude wetland ecosystems, although long-term field observations are required to evaluate ecosystem-scale consequences. Given the spatial heterogeneity and complex long-term succession of wetland ecosystems, future studies should combine long-term in situ monitoring with multifactorial experiments. These experiments should include independent incubator replication and separate sequencing of individual microcosm samples. Together, these approaches could help assess the reproducibility of associations observed in pooled samples and clarify how environmental variability and microbial processes jointly influence litter decomposition and nutrient cycling across spatial and temporal scales.

## Figures and Tables

**Table 1:** Annual decomposition rate constant (*k*) and temperature sensitivity (*Q*_10_) of single and mixed-litter treatments under different incubation temperatures.

Litter Type	Temperature Condition	Decomposition Constant *k* (yr^−1^)	Temperature Sensitivity *Q*_10_	*F*	*p*
CA	15 °C	1.53 ± 0.11 c	1.28 ± 0.05 b (15–25 °C)	*F*_2,6_ = 95.305	<0.001
	25 °C	1.96 ± 0.05 a	1.21 ± 0.01 a (25–35 °C)		
	35 °C	2.37 ± 0.05 b			
CL	15 °C	1.01 ± 0.06 c	1.85 ± 0.23 a (15–25 °C)	*F*_2,6_ = 200.487	<0.001
	25 °C	1.86 ± 0.12 a	1.25 ± 0.09 a (25–35 °C)		
	35 °C	2.31 ± 0.03 b			
ML	15 °C	1.58 ± 0.04 a	1.12 ± 0.14 b (15–25 °C)	*F*_2,6_ = 58.018	<0.001
	25 °C	1.76 ± 0.18 a	1.71 ± 0.10 b (25–35 °C)		
	35 °C	3.00 ± 0.24 b			
*k* ANOVA	Litter type	—		*F*_2,18_ = 23.806	<0.001
	Incubation temperature	—		*F*_2,18_ = 223.422	<0.001
	Litter type × incubation temperature	—		*F*_4,18_ = 14.922	<0.001
*Q *_10_ ANOVA	Litter type (15–25 °C)		—	*F*_2,6_ = 17.205	0.003
	Litter type (25–35 °C)		—	*F*_2,6_ = 41.240	<0.001

Values are presented as means ± standard deviations (SD; *n* = 3). Different lowercase letters indicate significant differences in *k* among incubation temperatures within a given litter treatment and in *Q*_10_ among litter treatments within a given temperature interval, according to Tukey’s HSD test (*p* < 0.05). The *F* and *p* values shown for *C. angustifolia* (CA), *C. lasiocarpa* (CL), and mixed litter (ML) represent the simple effects of incubation temperature within the respective litter treatments. For the *k* ANOVA, the *F* and *p* values represent the main effects of litter treatment and incubation temperature and their interaction. For the *Q*_10_ ANOVA, the *F* and *p* values represent the effect of litter treatment tested separately within the 15–25 °C and 25–35 °C intervals.

**Table 2:** Pearson correlation coefficients between decomposition constants and incubation temperature or initial litter stoichiometric ratios.

Factor	Decomposition Constant *k*	Decomposition Constant *k*_1_	Decomposition Constant *k*_2_
C/N	0.043	0.107	−0.017
C/P	−0.036	0.069	−0.126
N/P	−0.038	0.068	−0.129
Temperature	0.843 **	0.888 **	0.746 *

Asterisks denote significance levels: * *p* < 0.05, ** *p* < 0.01. C/N, carbon-to-nitrogen ratio; C/P, carbon-to-phosphorus ratio; N/P, nitrogen-to-phosphorus ratio. *k* represents the decomposition constant calculated over the entire 90-d decomposition period, whereas *k*_1_ and *k*_2_ represent the decomposition constants calculated for the 0–50-d and 50–90-d intervals, respectively.

**Table 3:** Topological properties of the bacterial co-occurrence network.

Temperature Condition	Node	Edge	Average Degree	Average Clustering Coefficient	Proportion of Positive Correlation/%
15 °C	60	204	6.8	0.555	93.14%
25 °C	60	147	4.9	0.583	92.52%
35 °C	64	300	9.4	0.690	97.67%

Nodes represent bacterial operational taxonomic units (OTUs), whereas edges represent significant pairwise correlations among OTUs. Average degree indicates the mean number of connections per node, and the average clustering coefficient reflects the tendency of neighboring nodes to form interconnected clusters. Positive correlations (%) denote the proportion of positive edges relative to the total number of edges in each network.

**Table 4:** Topological properties of the fungal co-occurrence network.

Temperature Condition	Node	Edge	Average Degree	Average Clustering Coefficient	Proportion of Positive Correlation/%
15 °C	63	97	3.079	0.382	80.41%
25 °C	47	96	4.085	0.518	82.29%
35 °C	32	71	4.438	0.660	92.96%

Nodes represent fungal operational taxonomic units (OTUs), whereas edges represent significant pairwise correlations among OTUs. Average degree indicates the mean number of connections per node, and the average clustering coefficient reflects the tendency of neighboring nodes to form interconnected clusters. Positive correlations (%) denote the proportion of positive edges relative to the total number of edges in each network.

**Table 5:** Results of three-way analysis of variance: Effects of incubation time, litter type, temperature, and their interactions on soil physicochemical properties.

Factors	df	Soil Organic Carbon		Total Nitrogen		Total Phosphorus		pH	
		F Value	*p*	F Value	*p*	F Value	*p*	F Value	*p*
Time (D)	2	8.83	<0.001	554.89	<0.001	772.53	<0.001	1677.31	<0.001
Litter type (L)	2	44.41	<0.001	79.72	<0.001	614.08	<0.001	119.13	<0.001
Temperature (T)	2	17.48	<0.001	42.94	<0.001	12.22	<0.001	585.57	<0.001
D × L	4	2.3	0.07	6.07	<0.001	343.01	<0.001	28.62	<0.001
D × T	4	3.08	0.023	176.4	<0.001	27.41	<0.001	149.91	<0.001
L × T	4	3.51	0.013	6.48	<0.001	2.77	0.036	22.71	<0.001
D × L × T	8	3.15	0.005	3.56	0.002	6.6	<0.001	16.94	<0.001

The effects of incubation time, litter type, temperature, and their interactions were evaluated using three-way analysis of variance (ANOVA). df, degrees of freedom. *p* < 0.05 and *p* < 0.01, representing significant and highly significant effects, respectively.

## Data Availability

The original contributions presented in this study are included in the article/Supplementary Material. Further inquiries can be directed to the corresponding authors.

## Supplementary Materials

The following supporting information can be downloaded at: https://www.mdpi.com/article/10.3390/microorganisms14102320/s1, Figure S1. Temporal variation in bacterial α-diversity during litter decomposition. Note: Violin plots show bacterial richness (Sobs, Chao1, and ACE) and diversity (Shannon and Simpson) indices calculated separately for 27 composite soil samples collected after 20, 50, and 90 d of decomposition. Each composite sample was formed by pooling soil subsamples from three microcosms within the same litter treatment × incubation temperature × harvest-time combination before DNA extraction. Each harvest-time group comprises nine community profiles representing the combinations of three litter treat-ments and three incubation temperatures. Black points and error bars represent means ± SD. Different lowercase letters indicate significant differences among decomposition times (*p* < 0.05). For each index, one-way ANOVA followed by Tukey’s HSD test was used when parametric assumptions were met; otherwise, Kruskal–Wallis tests followed by Dunn’s tests with Holm correction were applied; Figure S2. Temporal variation in fungal α-diversity during litter decomposition. Note: Violin plots show fungal richness (Sobs, Chao1, and ACE) and diversity (Shannon and Simpson) indices calculated separately for 27 composite soil samples collected after 20, 50, and 90 d of decomposition. Each composite sample was formed by pooling soil subsamples from three microcosms within the same litter treatment × incubation temperature × harvest-time combination before DNA extraction. Each harvest-time group comprises nine community profiles representing the combinations of three litter treat-ments and three incubation temperatures. Black points and error bars represent means ± SD. Different lowercase letters indicate significant differences among decomposition times (*p* < 0.05). For each index, one-way ANOVA followed by Tukey’s HSD test was used when parametric assumptions were met; otherwise, Kruskal–Wallis tests followed by Dunn’s tests with Holm correction were applied; Figure S3. Bootstrap significance of direct, indirect, and total effects in the partial least squares structural equation model (PLS-SEM). Note: Cells show two-sided bootstrap p values for standardized direct, indirect, and total effects from the same PLS-SEM presented in Figure 10 and Figure 11. The model was fitted using 27 treatment-level observations, with one observation per litter treatment × incuba-tion temperature × harvest-time combination. Soil properties and litter nutrient accumulation indices measured in the 81 micro-cosms were averaged across the three microcosms within each combination. Microbial variables were derived from the corre-sponding 27 composite soil samples, each formed by pooling soil subsamples from three microcosms before DNA extraction, and matched with the soil and litter averages by experimental combination. Statistical significance was assessed using 5000 bootstrap resamples. Columns represent predictors and rows represent response variables. Asterisks denote significance (*p* < 0.05, ** *p* < 0.01, *** *p* < 0.001), and shading indicates significance level. Blank cells indicate no corresponding effect, whereas em dashes indicate the same construct or a non-applicable comparison. *C. angustifolia* was the reference litter treatment for the two litter contrasts.

## Abbreviations

The following abbreviations are used in this manuscript:

CA	*Calamagrostis angustifolia*
CL	*Carex lasiocarpa*
ML	Mixed litter
SOC	Soil organic carbon
TN	Total nitrogen
TP	Total phosphorus
LTOC	Litter total organic carbon
NAI	Nutrient accumulation index
Q_10_	Temperature sensitivity coefficient
OTU	Operational taxonomic unit
ASV	Amplicon sequence variant
RDA	Redundancy analysis
SEM	Structural equation modeling
PLS-SEM	Partial least squares structural equation modeling
