# Supplementary material for: Simulated Warming Alters Soil Nutrient Dynamics Through Microbial Community Shifts During Wetland Plant Litter Decomposition
Source: Microorganisms. 2026 Oct 12;14(10):2320. doi: 10.3390/microorganisms14102320 (PMC13707877; doi:10.3390/microorganisms14102320)
Supplement: Supplementary file 1 [file microorganisms-14-02320-s001.zip › microorganisms-4601673-supplementary.pdf]

Supplementary Figures:

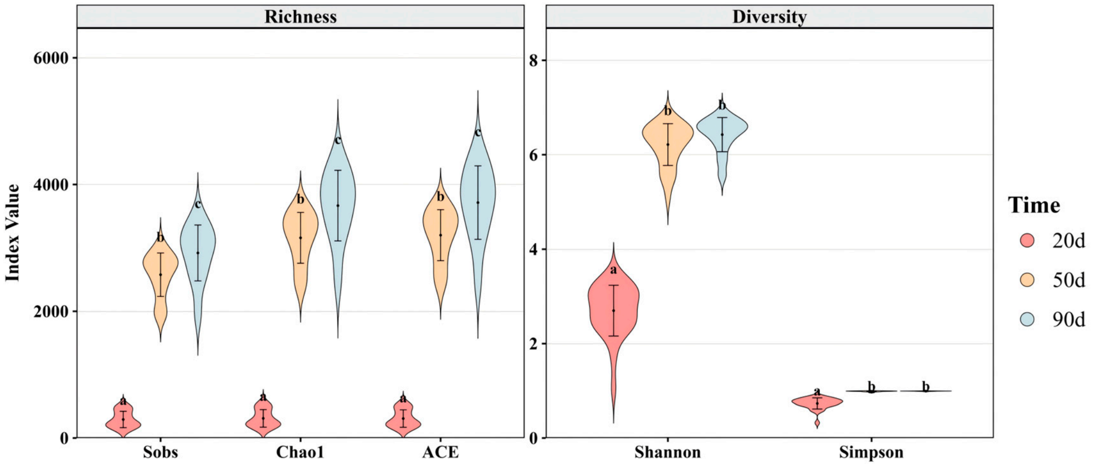

Figure S1-Temporal variation in bacterial α-diversity during litter decomposition.

Note: Violin plots show bacterial richness (Sobs, Chao1, and ACE) and diversity (Shannon and Simpson) indices calculated separately for 27 composite soil samples collected after 20, 50, and 90 d of decomposition. Each composite sample was formed by pooling soil subsamples from three microcosms within the same litter treatment × incubation temperature × harvest-time combination before DNA extraction. Each harvest-time group comprises nine community profiles representing the combinations of three litter treat-ments and three incubation temperatures. Black points and error bars represent means ± SD. Different lowercase letters indicate significant differences among decomposition times ($P < 0.05$). For each index, one-way ANOVA followed by Tukey's HSD test was used when parametric assumptions were met; otherwise, Kruskal–Wallis tests followed by Dunn's tests with Holm correction were applied.

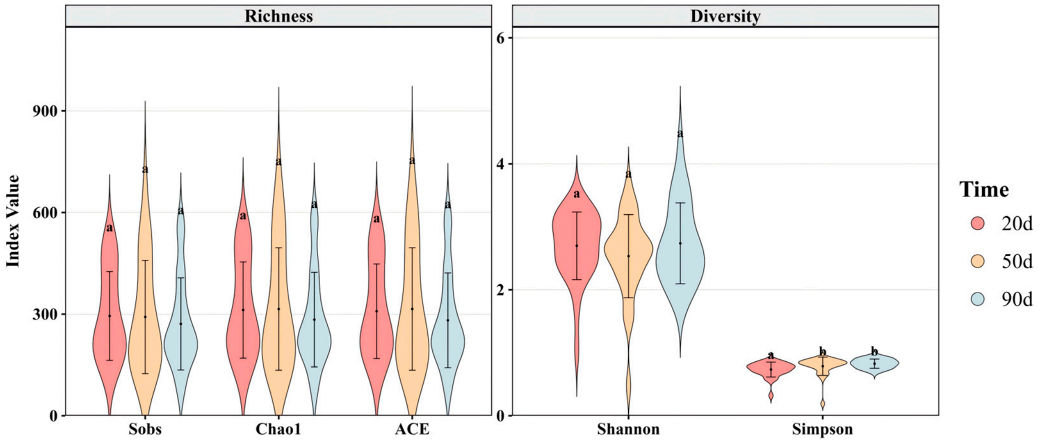

Figure S2-Temporal variation in fungal α-diversity during litter decomposition.

Note: Violin plots show fungal richness (Sobs, Chao1, and ACE) and diversity (Shannon and Simpson) indices calculated separately for 27 composite soil samples collected after 20, 50, and 90 d of decomposition. Each composite sample was formed by pooling soil subsamples from three microcosms within the same litter treatment × incubation temperature × harvest-time combination before DNA extraction. Each harvest-time group comprises nine community profiles representing the combinations of three litter treat-ments and three incubation temperatures. Black

points and error bars represent means ± SD. Different lowercase letters indicate significant differences among decomposition times (P < 0.05). For each index, one-way ANOVA followed by Tukey's HSD test was used when parametric assumptions were met; otherwise, Kruskal–Wallis tests followed by Dunn's tests with Holm correction were applied.

Response variables	Incubation temperature	Decomposition time	C. lasiocarpa vs C. angustifolia	Mixed litter vs C. angustifolia	Soil pH	Microbial alpha-diversity	C-N functional-module axis	itter decompositio nutrient axis	Soil C-N-P axis	
Incubation temperature	—									Direct effect
Decomposition time		—								Direct effect
C. lasiocarpa vs C. angustifolia			—							Direct effect
Mixed litter vs C. angustifolia				—						Direct effect
Soil pH					—					Direct effect
Microbial alpha-diversity	0.038*	0.052	0.282	0.131	0.023*	—				Direct effect
C-N functional-module axis	0.793	0.791	0.928	0.759	0.607	0.627	—			Direct effect
Litter decomposition-nutrient axis						0.919	0.955	—		Direct effect
Soil C-N-P axis						0.917	0.832	0.973	—	Direct effect
Incubation temperature	—									Indirect effect
Decomposition time		—								Indirect effect
C. lasiocarpa vs C. angustifolia			—							Indirect effect
Mixed litter vs C. angustifolia				—						Indirect effect
Soil pH					—					Indirect effect
Microbial alpha-diversity						—				Indirect effect
C-N functional-module axis	0.630	0.637	0.661	0.622	0.623		—			Indirect effect
Litter decomposition-nutrient axis	0.927	0.944	0.955	0.975	0.907	0.890		—		Indirect effect
Soil C-N-P axis	0.843	0.939	0.969	0.667	0.730	0.999	0.967		—	Indirect effect
Incubation temperature	—									Total effect
Decomposition time		—								Total effect
C. lasiocarpa vs C. angustifolia			—							Total effect
Mixed litter vs C. angustifolia				—						Total effect
Soil pH					—					Total effect
Microbial alpha-diversity	0.038*	0.052	0.282	0.131	0.023*	—				Total effect
C-N functional-module axis	0.996	0.574	0.915	0.961	0.817	0.627	—			Total effect
Litter decomposition-nutrient axis	0.927	0.944	0.955	0.975	0.907	0.917	0.955	—		Total effect
Soil C-N-P axis	0.843	0.939	0.969	0.667	0.730	0.900	0.789	0.973	—	Total effect

Bootstrap significance

ns $P \geq 0.05$ * $P < 0.05$ ** $P < 0.01$ *** $P < 0.001$

Cells show two-sided bootstrap P values for standardized direct, indirect, and total effects from the same PLS-SEM model used in Figures 10 and 11 (5,000 resamples). * P < 0.05; ** P < 0.01; *** P < 0.001. CL-CA and ML-CA denote li

Figure S3-Bootstrap significance of direct, indirect, and total effects in the partial least squares structural equation model (PLS-SEM).

Note: Cells show two-sided bootstrap P values for standardized direct, indirect, and total effects from the same PLS-SEM presented in Figures 10 and 11. The model was fitted using 27 treatment-level observations, with one observation per litter treatment × incuba-tion temperature × harvest-time combination. Soil properties and litter nutrient accumulation indices measured in the 81 micro-cosms were averaged across the three microcosms within each combination. Microbial variables were derived from the corre-sponding 27 composite soil samples, each formed by pooling soil subsamples from three microcosms before DNA extraction, and matched with the soil and litter averages by experimental combination. Statistical significance was assessed using 5,000 bootstrap resamples. Columns represent predictors and rows represent response variables. Asterisks denote significance (P < 0.05, ** P < 0.01, *** P < 0.001), and shading indicates significance level. Blank cells indicate no corresponding effect, whereas em dashes indicate the same construct or a non-applicable comparison. C. angustifolia was the reference litter treatment for the two litter contrasts
